# Social and biological pathways linking relationships with companion animals to human well-being

**DOI:** 10.3389/fpsyg.2026.1810355

**Published:** 2026-09-03

**Authors:** Hikari Koyasu, Sakura Ogasawara, Eiji Miyauchi, Kaede Tsuda, Takefumi Kikusui, Miho Nagasawa

**Affiliations:** 1 Laboratory of Human-Animal Interaction and Reciprocity, Department of Animal Science and Biotechnology, Azabu University, Sagamihara, Kanagawa, Japan; 2 Institute for Molecular and Cellular Regulation, Gunma University, Maebashi, Japan

**Keywords:** companion animal, human–animal interaction, mental health, social interaction, well-being, cortisol, gut microbiota

## Abstract

**Introduction:**

Companion animals are widely recognized for their potential to support human mental health and well-being. These benefits may arise through changes in social relationships and physiological processes. This study examined whether dog and cat ownership and attachment promote closer social relationships and higher well-being, the relative magnitude of direct and indirect effects of companion animals on well-being, and whether physiological factors, including cortisol concentration and gut microbiota composition, are involved in these processes.

**Methods:**

Two complementary studies were conducted. Study 1 used the Experience Sampling Method (ESM) to record daily social interactions over 2 weeks. Participants also completed questionnaires assessing pet ownership, attachment, social attitudes (including the Cultural Estrangement Inventory), self-esteem, general trust, and well-being. Structural equation modeling was used to examine direct and indirect pathways from human–animal relationships to well-being. Study 2 examined physiological correlates by analyzing urinary basal cortisol concentrations and gut microbiota composition.

**Results:**

In Study 1, dog ownership was associated with closer relationships with others. Family interaction was associated with well-being through two pathways: when confined to the household, it was associated with lower self-esteem and well-being, whereas when accompanied by interactions with the local community and friends, it was associated with higher self-esteem and well-being. These indirect effects were particularly pronounced among female participants, while no significant direct effects of companion animals on well-being were observed. In Study 2, lower cortisol concentrations were associated with greater daily conversation with close others, and gut microbiota profiles differed according to dog ownership and the frequency of family social contact.

**Discussion:**

These findings suggest that companion animals may contribute to human well-being through interconnected social and biological pathways. Dogs may promote well-being not simply by strengthening family relationships, but by facilitating broader social connections beyond the household. The findings also suggest that physiological processes, including cortisol regulation and gut microbiota composition, may be involved in the relationship between human–animal interactions and well-being.

## 1 Introduction

Companion animals, particularly dogs and cats, are increasingly believed to be important contributors to human well-being. A growing body of research suggests that interactions with companion animals can buffer stress, reduce loneliness, and provide emotional support (Beetz et al., 2012). However, systematic reviews examining associations between pet ownership and loneliness or social isolation have shown that existing evidence remains insufficient to demonstrate consistent effects (Kretzler et al., 2022; Gilbey and Tani, 2015). Similarly, findings on the relationship between pet ownership and mental health outcomes have been mixed, including positive, negative, and null effects (Herzog, 2011; Brooks et al., 2018; Scoresby et al., 2021; Zilcha-Mano et al., 2011).

These inconsistencies suggest that the effects of companion animals on human well-being are shaped by multiple moderating factors, including individual characteristics of owners, life circumstances, interaction styles, and caregiving demands (Charmaraman et al., 2022; Kurdek, 2008; Northrope et al., 2025; Spitznagel et al., 2017). Accordingly, it may not be pet ownership per se, but rather the quality and nature of the human–animal relationship, that determines whether companion animals exert beneficial effects on well-being.

Beyond dyadic human–animal relationships, companion animals may also influence well-being indirectly by shaping social relationships among humans (Koyasu et al., 2023). Dogs, in particular, have been shown to function as social catalysts by encouraging outdoor activity, increasing opportunities for social interactions, and fostering perceptions of social support and trust (Bulsara et al., 2007; Liu et al., 2019; Wood et al., 2005; Wood et al., 2015; Ishiguro et al., 2025; Koohsari et al., 2021). Cats, by contrast, are more often associated with emotional support within the home and with the quality of close or family relationships (Stammbach and Turner, 1999). Dog ownership is expected to influence well-being through interactions with both family members and the local community, whereas cat ownership is expected to influence well-being primarily through interactions within the family, with more limited influence from community-level interactions. These species-specific patterns suggest that companion animals may contribute to human well-being through distinct social pathways.

Previous studies have reported gender differences in attitudes toward companion animals, caregiving involvement, and the psychological correlates of pet ownership, with women generally showing stronger emotional attachment and greater involvement in daily pet care than men (Herzog, 2011; Power, 2008). Gender differences have also been observed in the structure of social networks, with women tending to derive greater psychological benefits from close family and community relationships. Based on these findings, we hypothesized that the pathways linking companion animals to well-being would differ by gender. Specifically, we expected that men would be more likely to benefit through broader external social networks (e.g., neighbors and friends), whereas women would be more likely to benefit through family and community relationships embedded in daily life.

Understanding the social pathways through which dog and cat ownership may influence well-being via family and community relationships is important. Social relationships are among the strongest predictors of health and mortality (Holt-Lunstad et al., 2010). Poor relationship quality and lack of social support have been linked to increased risk of depression (Kawachi and Berkman, 2001; Teo et al., 2013), whereas social connectedness is associated with better psychological and physical health outcomes (Holt-Lunstad, 2017). Clarifying how companion animals influence human social relationships is therefore essential for understanding their potential contributions to human well-being.

Despite this importance, much of the existing literature has relied on retrospective self-report measures that require participants to summarize past experiences, which may limit the accuracy with which everyday social interactions are assessed (Kahneman et al., 2004). Although some studies have employed objective approaches, such as neural or physiological measures, these methods are typically conducted in controlled settings and may not fully capture the frequency, context, and immediacy of daily social contacts in naturalistic environments. Moreover, it remains difficult to disentangle direct effects of interactions with animals from indirect effects mediated through human social relationships in everyday life. To address this issue, the present study employed the Experience Sampling Method (ESM), which allows for near real-time assessment of social interactions and minimizes recall bias. ESM is particularly well-suited for examining dynamic associations between daily social experiences and well-being.

In parallel, accumulating evidence indicates that biological mechanisms may play a role in the relationship between companion animals and human well-being. Interactions with dogs, especially those involving physical contact, have been associated with changes in physiological indicators related to stress regulation and social bonding, including cortisol secretion, autonomic nervous system activity, and oxytocin release (Cole et al., 2007; Handlin et al., 2011; Nagasawa et al., 2015). Cortisol is an established biomarker of stress and activity of the hypothalamic–pituitary–adrenal (HPA) axis and has been widely used in studies investigating the physiological effects of human-animal interactions. Therefore, this serves as a useful indicator for assessing stress-related changes associated with interactions with companion animals.

In addition to endocrine responses, recent research has emphasized the role of the gut–brain axis, a bidirectional communication system linking the central nervous system and the gastrointestinal tract (Cryan and Dinan, 2012). Gut microbiota is known to play a key role in regulating stress responses, emotional states, and overall physiological homeostasis. Emerging evidence also suggests that companion animal ownership may be associated with differences in gut microbiota composition (Azad et al., 2013; Song et al., 2013; Tun et al., 2017), reflecting lifestyle and environmental factors linked to human–animal interactions. Our recent research further demonstrated that the gut microbiota of children who own dogs may enhance prosocial behaviors (Miyauchi et al., 2025). Together, these findings suggest that gut microbiota may function as a potential mediator of psychological and social health through the gut–brain axis. Nevertheless, few studies have attempted to integrate these physiological indicators with social and psychological processes within a unified framework.

The aim of this study was to investigate how companion animals contribute to human well-being through social and physiological pathways. Specifically, this study addressed three research questions: (1) whether dog and cat ownership and attachment are associated with closer relationships with significant others, and whether such relationships are linked to higher well-being; (2) the relative magnitude of direct and indirect effects of companion animals on well-being; and (3) whether physiological factors, including cortisol concentration and gut microbiota composition, are involved in these processes.

Based on previous findings, we hypothesized that companion animal ownership would influence well-being indirectly through social relationships. We further predicted that the pathways linking companion animal ownership and well-being would differ according to the species owned. Specifically, dog ownership would be associated with higher well-being through both family relationships and community or friendship networks, whereas cat ownership would be associated primarily with family relationships. We also predicted that these pathways would differ by gender. Men would be more likely to benefit through external social networks, such as neighbors and friends, particularly in relation to dog ownership, whereas women would be more likely to benefit through family relationships regardless of whether they owned dogs or cats.

To address these questions, the present study adopted a multilayered approach to examine how interactions with companion animals are linked to human well-being through interconnected social and biological pathways. Study 1 used ESM data and structural equation modeling (SEM) to examine the direct and indirect associations among companion animal ownership, social relationships, and well-being. Study 2 explored potential physiological mechanisms underlying these associations by examining cortisol concentrations and gut microbiota characteristics.

## 2 Methods

### 2.1 Participants

Participants were required to be at least 10 years old. Participants included a broad age range enabling a more comprehensive assessment of household dynamics and social interactions. The lower age threshold of 10 years was selected because participants were required to use a smartphone to complete the study assessments and provide data. Both pet owners and non-pet owners were recruited. Informed consent was obtained from all participants. For participants under 18 years of age, parental consent was also obtained. Recruitment occurred through Azabu University, Aoyama Gakuin University, and a research participant recruitment website.^1^ Participants received a 5,000-yen Amazon gift card as compensation, as stated in the recruitment materials. A total of 36 individuals were excluded due to withdrawal or incomplete data, resulting in a sample of 184 participants whose data was included in the final dataset.

### 2.2 Procedure

Data collection consisted of an online ESM survey for 2 weeks, a supplemental questionnaire, and the collection of urine and fecal samples. During the ESM period, participants collected urine and fecal samples at home and completed an online questionnaire on the final day. Participants provided daily reports on the frequency of their interactions with family, neighbors, and friends, as well as with their pets, and on their sleep duration and number of meals. After the two-week ESM program ended, they filled out a supplemental questionnaire the next day. The final questionnaire included basic information such as gender, age; number of family members; general trust, cultural estrangement inventory, well-being over the past 2 weeks, self-esteem, everyday discrimination scale, the big five personality traits, and factors considered to affect the gut microbiota. In addition, it also included attachment to their pets for pet owners.

### 2.3 Assessment of interaction frequency

Participants’ interaction frequency with family members, community members, and pets was assessed using EXKUMA, a smartphone-based ESM platform. Participants completed a brief questionnaire (approximately 5 min) once daily during the 2 weeks, reporting on interactions from the previous day. Questionnaire items were adapted from Inaba (2016) and captured interactions with family, friends, local residents, and dogs/cats, as well as behaviors related to meals and sleep.

#### 2.3.1 Interaction with family members

Participants reported whether they had conversations or shared space, or activities with family members. For the item “Yesterday, did you have any conversations with your family beyond simple greetings?” response options included father, mother, siblings, grandparents, partner, children, no conversations with family, or other. If “other” was selected, participants provided additional details in a free-text field. In subsequent analyses, the proportion of days on which participants conversed with each specific person (e.g., conversation_mother, conversation_father, etc.) was calculated, as well as the overall proportion of days on which participants conversed with any family member (Family Conversation). Participants were also asked the number of family members with whom they watched television or streaming content (referred to as “TV”), went shopping (referred to as “Shopping”), dined out or ate meals at home (referred to as “Eating”). A value of 10 indicated participation with 10 or more people. These values were adjusted for the number of family members, by calculating the average amount of conversation or activities for 2 weeks by the number of family members to calculate the amount of conversation per family member.

#### 2.3.2 Interaction with community members

For the item “Yesterday, how many people did you interact with in the following situations?,” participants reported the number of individuals with whom they “exchanged greetings (referred to as “Greeting Neighbors,”)” “had interactions beyond greetings (referred to as “Neighborhood Interaction,”)” “interacted through lessons or hobbies (referred to as “Hobbies,”)” “interacted in volunteer or community service activities,” and “interacted through their work, including part-time jobs (referred to as “Job.”)” Responses were recorded on a 0–10 scale, with 10 indicating interactions with 10 or more people. Participants also reported interactions with close friends, specifying the number of individuals they “contacted via computer or phone (referred to as “Digital Friend Contact: DFC”)” and “met in person (referred to as “Out of School Interaction”),” using the same scale.

#### 2.3.3 Interaction with pets

Interactions with pets (dogs and cats) were assessed via two items: (1) “time spent in physical contact with the pet inside the home” and (2) “time spent on pet care,” measured on a 6-point scale: not owning a pet, 0 min, <15 min, <30 min, 30 min–1 h, and >1 h. Additional items assessed interactions with nonfamily members mediated by the participant’s pet, as well as interactions with other people’s pets, using a 5-point scale: 0 min, <15 min, <30 min, 30 min–1 h, and >1 h.

### 2.4 Additional daily measures

Participants reported on diet and sleep, factors potentially influencing daily well-being. For meals, options included no meals, one meal, two meals, or three meals. Sleep duration was reported using a slider, 0–10 h, with 10 indicating >10 h. Participants were also asked the open-ended question: “Where are you and what are you doing right now as you answer this?”

### 2.5 Calculation of variables related to family and community interactions

Interactions with Family and Community: For the number of conversations with family members (father, mother, siblings, grandparents, partner, and children), we calculated, for participants cohabiting with each target family member, the percentage total number of days on which conversations occurred during the 2 weeks. For each variable related to interactions with family and community, we computed the total number of individuals with whom participants interacted over the same period.

### 2.6 Final questionnaire

On the final day of the ESM survey, participants completed an online questionnaire administered via Google Forms.

#### 2.6.1 Descriptive statistics and demographics

Participants reported gender (female, male, or other), age, occupation (elementary school student, junior high school student, high school student, undergraduate or graduate student, other student, employed, or other), number of cohabiting family members, household composition (father, mother, siblings, grandparents, partner, children, or other), and the number of close friends they felt comfortable with (1–10+). Regarding pet ownership, participants indicated past and current experiences with pets, selecting “no experience of pet ownership,” “experience of keeping dogs or cats,” or “experience of keeping other types of pets.” Only participants reporting experience with dogs or cats were asked for further details: pet type (dog or cat), ownership period (past or current), specific breed, keeping environment (free access indoors, restricted indoor area, or outdoors), and any problematic behaviors or difficulties in daily life. Participants who reported currently owning a dog (cat) were classified as dog (cat) owners, whereas all other participants were classified as non-owners. Participants who owned both a dog and a cat were included in both owner groups.

#### 2.6.2 Attachment to pets

Participants who had experience keeping dogs, cats, or other pets reported their attachment to pets using the 12-item scale developed by Kaneko (2018). Responses were rated on a five-point Likert scale ranging from 1 (strongly disagree) to 5 (strongly agree). Sample items are presented in Supplementary Table S1. Kaneko (2018) conceptualized attachment to pets as comprising two dimensions: basic attachment, reflecting affectionate feelings and comfort derived from pets, and close attachment, reflecting strong psychological closeness and dependence on pets. Following this framework, six items were used to calculate basic attachment scores and six items were used to calculate close attachment scores. Sum scores were calculated for each dimension, with higher scores indicating stronger attachment.

#### 2.6.3 General trust

General trust was assessed using the six-item scale developed by Yamagishi and Yamagishi (1994). Items included: “Most people are basically honest,” “I tend to trust others,” “Most people are basically good and kind,” “Most people trust others,” “Most people are trustworthy,” and “Most people will trust others in return when they are trusted.” Participants rated each item on a 7-point Likert scale ranging from 1 (strongly disagree) to 7 (strongly agree). These sums were calculated as a measure of general trust.

#### 2.6.4 Cultural estrangement inventory

A modified version of the Cultural Estrangement Inventory (CEI) developed by Cozzarelli and Karafa (1998) was used to assess tendencies toward cultural estrangement. In previous studies, items referred to broad cultural affiliations, such as “country” or “society.” In the present study, to assess perceived value congruence with more immediate communities, such as family, local community, and friends, all references to cultural affiliation were replaced with “people around me.” The 10-item scale included: “I feel that my opinions on important matters are typical or similar to those of people around me,” “I feel that my worldview matches that of people around me,” “I feel that many of my values are universal and shared by people around me,” “I feel that I am a good example of what people around me consider a typical Japanese person,” “I strongly identify with the values of people around me,” “I feel that most people around me do not understand me,” “My opinions on important matters tend to differ from those of people around me,” “I feel very different from people who are called ‘ordinary,’” “I sometimes feel that I do not fit in well with people around me,” and “I am sometimes told by people around me that I am unusual.” Responses were rated on a 7-point Likert scale ranging from 1 (strongly disagree) to 7 (strongly agree). Based on the original research (Cozzarelli and Karafa, 1998), CEI scores were calculated and analyzed using the total score as well as the Atypical and Misfit subscale scores.

#### 2.6.5 Well-being

Mental health status was assessed using the Japanese version of the WHO-5 Well-Being Index (Awata et al., 2007). The scale consists of five items: “I have felt cheerful and in good spirits,” “I have felt calm and relaxed,” “I have felt active and vigorous,” “I woke up feeling fresh and rested,” and “My daily life has been filled with things that interest me.” Participants reported how often they had experienced each state over the past 2 weeks using a 6-point scale ranging from 0 (at no time) to 5 (all of the time). For analysis, total scores for all items were used.

#### 2.6.6 Self-esteem

Self-esteem, defined by Rosenberg (1965) as a global self-concept and overall evaluation of one’s own worth rather than the presence or absence of specific attributes or abilities, was assessed using the Japanese version of the Rosenberg Self-Esteem Scale (Mimura and Griffiths, 2007). The scale comprises 10 items designed to capture both positive and negative self-attitudes. Sample items include: “On the whole, I am satisfied with myself,” “At times, I think I am no good at all,” “I feel that I have several good qualities,” and “I can do things as well as most other people.” For analysis, total scores for all items were used.

#### 2.6.7 Everyday discrimination scale

To assess participants’ connection to the public social world, the frequency of relatively minor but chronic experiences of everyday discrimination was assessed using the Everyday Discrimination Scale (Williams et al., 1997). Participants reported how often they experienced the following five situations: (1) being treated with less courtesy or respect than other people; (2) receiving poorer service than other people at restaurants or stores; (3) experiencing behaviors that suggest others think they are not smart; (4) experiencing behaviors that suggest others are afraid of them; and (5) being threatened or harassed. Responses were rated on a 6-point scale: 0 = never, 1 = less than once a year, 2 = a few times a year, 3 = a few times a month, 4 = at least once a week, and 5 = almost every day. Participants were also asked to indicate perceived reasons for these discriminatory experiences by selecting all that applied from the following options: ancestry or national origin, gender, race, age, religion, height, weight, other aspects of physical appearance, sexual orientation, level of education or income, physical disability, skin color, ethnicity, no experience, and other. This scale was not included in this study.

#### 2.6.8 Big-five personality traits

Personality traits were assessed using the Japanese version of the Ten Item Personality Inventory (TIPI-J; Oshio et al., 2014), which measures the Big Five dimensions: Extraversion, Agreeableness, Conscientiousness, Neuroticism, and Openness to Experience. The TIPI-J consists of 10 items rated on a 7-point Likert scale. Scores for each personality dimension were calculated according to the standard scoring procedure and used in subsequent analyses.

#### 2.6.9 Items considered to affect the gut microbiota

Participants provided urine and fecal samples and reported factors potentially influencing gut microbiota. These factors included: (1) dietary intake: supplements, lactic acid bacteria beverages, and yogurt in the week before fecal sampling (type, amount, and frequency); (2) medication use: including antibiotics within 6 months before fecal sampling (type, amount, and frequency); (3) lifestyle behavior: frequency of smoking and alcohol consumption; (4) medical history: disease name, timing, and symptoms; and (5) body mass index (BMI).

### 2.7 Urine and fecal sample collection

To investigate associations between mental health, interactions with others and pets, and physiological indices, basal cortisol levels and gut microbiota were analyzed. Cortisol was measured in the first morning urine, and gut microbiota were assessed using fecal samples collected immediately after defecation. Collection materials (e.g., droppers and tubes) were mailed to participants’ homes before the start of the survey. During the two-week questionnaire period, participants collected urine samples three times and a fecal sample once. Participants were instructed to: (1) avoid sampling after unusually large changes in physical activity or mood; (2) avoid sampling during menstruation; and (3) collect samples in the early morning on a convenient day. Approximately 12 mL of urine was collected per sample, with the collection date recorded. Samples were stored in participants’ home freezers until shipment. Fecal samples were self-collected using RNAlater™ Stabilization Solution and stored at room temperature. All samples were transferred to a − 80 °C freezer within 1 week of collection.

### 2.8 Hormone assay

Urinary cortisol concentration was measured as an index of hormonal activity using an enzyme immunoassay.

Plate Coating: A secondary antibody (Mouse IgG-Fc Fragment Antibody; BET A90-131A) was diluted 1:500 in carbonate buffer and dispensed at 100 μL per well in a 96-well high-binding plate (EIA/RIA plate; COSTER, Cat. No. 9018). Plates were incubated overnight at room temperature for antibody adsorption. The following day, the carbonate buffer was removed, 200 μL of assay buffer was added per well, and plates were stored at 4 °C.

Concentration Measurement: After removing the assay buffer, wells were washed three times using a plate washer (Bio-Rad Laboratories; Model 1,575 ImmunoWash). Standard cortisol solutions (1 μg/μL in methanol) were serially diluted 2-fold from 800 to 1.56 ng/mL; control samples, diluted urine samples were at 15 μL per well. Next, primary antibody (Anti-Cortisol antibody, ab1949; Abcam; 2 mg/mL diluted 1:200,000) and HRP conjugate (Cortisol-3-CMO-HRP; Cosmo Bio FKA403; 200 μg/20 μL/1 mL [×50]) were added at 100 μL per well. Plates were protected from light with aluminum foil and incubated at 4 °C for ≥6 h.

After incubation, wells were washed four times, and 150 μL of substrate buffer (prepared by mixing Substrate Buffer A and B) was added. Plates were incubated at 25 °C–30 °C for 5–20 min under light protection to allow color development. The reaction was stopped by adding 50 μL of 4 N sulfuric acid per well, yielding a yellow color. Absorbance was measured at 450 nm using a microplate reader (Model 680XR; Bio-Rad Laboratories). Cortisol concentration (ng/mg) was calculated from a standard curve and corrected for creatinine before further analyses.

### 2.9 Gut microbiota analysis

Gut microbiota were analyzed using 16S rRNA gene sequencing. Before DNA extraction, fecal samples underwent enzymatic treatment with lysozyme, achromopeptidase, and proteinase K. DNA was purified using phenol/chloroform/isoamyl alcohol extraction. The V3–V4 region of the 16S rRNA gene was PCR-amplified with universal primers 341F/805R. Amplicons were indexed, and libraries were prepared. Library quantity and quality were assessed using the KAPA Library Quantification Kit and Agilent TapeStation. Paired-end sequencing (2 × 300 bp) was conducted on the Illumina MiSeq platform. Participants with underlying medical conditions or a history of antibiotic use were excluded from microbiota analyses.

### 2.10 Statistical analysis

Sample size adequacy was evaluated based on commonly cited recommendations. Although various perspectives exist, a rule of thumb suggests approximately five observations per estimated parameter (Bentler and Chou, 1987). In addition, previous studies have suggested that sample sizes of approximately 100–200 or more are generally acceptable depending on model complexity (e.g., Kline 2016; Wolf et al., 2013). The present sample size (n = 184) falls within these recommended ranges and was considered adequate for the analysis.

#### 2.10.1 Preliminary analysis for structural equation Modeling (SEM)

As a preprocessing step to identify a better-fitting model for the structural equation modeling (SEM) analysis, group comparisons using the Wilcoxon rank-sum test and correlation analyses using Spearman’s rank correlation coefficient were conducted. The analyses included pet ownership status (dog and cat ownership), interaction variables related to pets, family, and the local community, and indicators of mental health that were considered as factors in the SEM. Based on the results of these preliminary analyses, the SEM model was constructed. All results from the preprocessing analyses are provided in the Supplementary materials.

##### 2.10.1.1 Study 1

Hypothesized models were tested using SEM. All behavioral variables were standardized before estimation. For the CEI, strong correlations were observed among the Atypical, Misfit, and Total scores, with similar patterns observed with other indicators; therefore, only the Total score was used in SEM as the index of cultural estrangement. Hypothesized models included both direct and indirect paths to examine the effects of current dog and cat ownership and modes of interaction with pets on mental health, with latent variables representing family (defined as Family Interaction) and community interactions (defined as Non-family Interaction, including interaction with local community and friends) as mediators. Indicators of pet interaction included: (1) average physical contact time with pets over 2 weeks (ESM), (2) average time spent caring for pets over 2 weeks (ESM), (3) basic attachment score toward pet (final-day questionnaire), and (4) dependent/close attachment score toward pets (final-day questionnaire). Gender differences have been reported in family/community interaction frequencies and mental health. Therefore, models using current dog/cat ownership as predictors were estimated separately by gender. In contrast, models using attachment and interaction-style variables as predictors were restricted to dog/cat owners. To avoid substantial sample-size imbalance, these models were estimated without stratification by gender or species.

##### 2.10.1.2 Study 2

To examine associations with physiological indices, urinary cortisol concentrations and gut microbiota characteristics were analyzed. For cortisol analyses, generalized linear mixed models were fitted with urinary cortisol concentration as the dependent variable and dog ownership, cat ownership, age, and gender as fixed effects. Cortisol concentrations were averaged across the three sampling days and log-transformed prior to analysis. To control for age and gender, each variable was regressed on these covariates, and partial correlations between residuals were calculated to examine associations among family and community interaction frequencies, mental health indices, and cortisol concentrations. To account for multiple comparisons, *p*-values were adjusted using the Benjamini–Hochberg procedure.

For gut microbiota analyses, beta diversity was visualized using principal coordinate analysis (PCoA) based on weighted UniFrac distances, and between-group differences were evaluated using PERMANOVA (adonis). Factors associated with microbial community structure were examined using distance-based redundancy analysis (db-RDA) and envfit. To assess the independent contributions of dog ownership and behavioral factors, variance partitioning was performed using partial RDA. Differential abundance at the taxon level was assessed using ANCOMBC2, adjusting for age, gender, BMI, and behavioral factors, with false discovery rate correction for multiple testing. For both Study 1 and Study 2, correlation analyses, group comparisons, and structural equation modeling (SEM) were conducted using JMP Student Edition 19(SAS Institute Inc., Cary, NC, United States). Analyses of gut microbiota data in Study 2 were performed in R (version 4.5.1) using the DADA2 and phyloseq packages.

## 3 Results

### 3.1 Descriptive statistics

Basic information of participants are shown in Table 1. The sample included both male and female participants spanning a broad age range and diverse occupational backgrounds. Participants differed in their living arrangements, social relationships, and pet ownership histories, including current and former dog and cat owners as well as individuals without pet ownership experience.

**Table 1:** Descriptive statistics of participant demographics, social relationships, and pet ownership.

Variable	Category	*N* (*n* = 184)
Sex	Male	60
	Female	124
Pet ownership	Dogs or cats	99
	Other animals	49
	Non-pet owner	36
Dog or cat ownership status*	Currently owns a dog	61
	Currently owns a cat	22
	Previously owned a dog	24
	Previously owned a cat	11
Family number living with	0	11
	1	46
	2	58
	3	49
	4	17
	5	3
Family member	Father	92
	Mother	114
	Siblings	59
	Grand parents	13
	Partner	49
	Children	33
	Other	3

Asterisk shows multiple responses were allowed for dog and cat ownership status.

### 3.2 Study 1: associations among pet ownership, social relationships, and well-being: SEM

**Figure 1:**
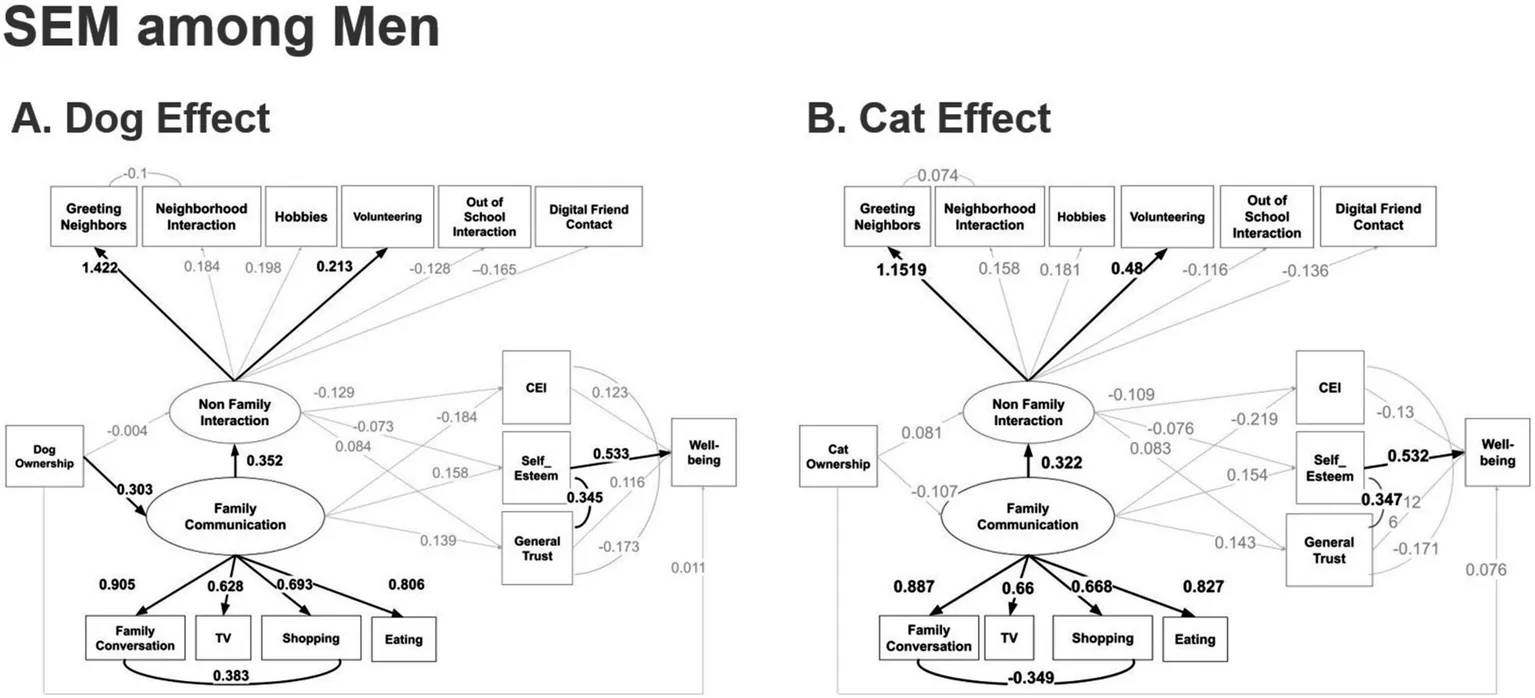
A model of the effects of pet ownership on well-being among men. **A** showed the dog effect and **B** showed the cat effect. The values in the figure represent standardized regression coefficients, factor loadings, or covariances. Black path indicates significant paths and light gray path indicates nonsignificant paths.

**Figure 2:**
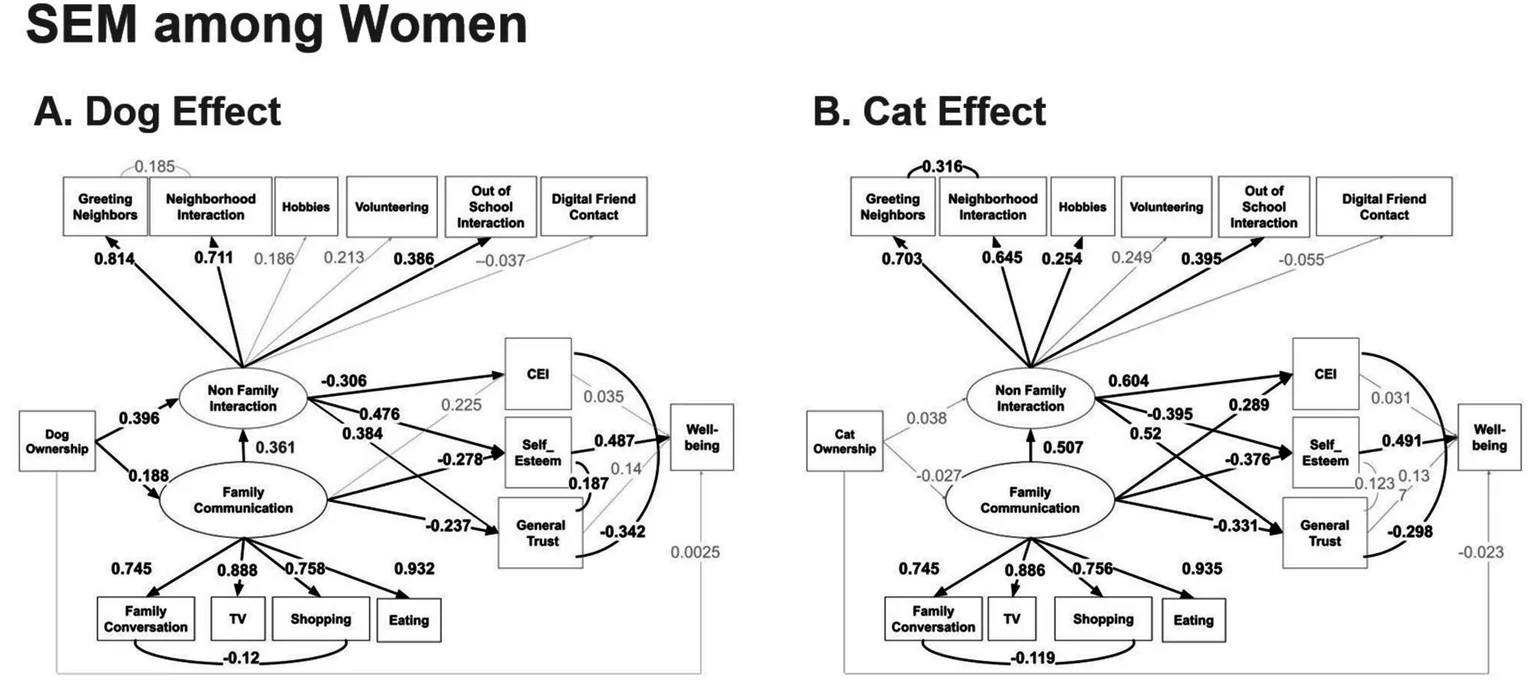
A model of the effects of pet ownership on well-being among women. **A** showed the dog effect and **B** showed the cat effect. The values in the figure represent standardized regression coefficients, factor loadings, or covariances. Black path indicates significant paths and light gray path indicates nonsignificant paths.

Figures present the results of the SEM examining the associations among pet ownership, social interactions, psychosocial variables, and well-being, analyzed separately for men (Figure 1) and women (Figure 2).

#### 3.2.1 Effects of current dog and cat ownership among men

For men, model fit indices indicated limited overall fit for both the dog-ownership model (Figure 1A; CFI = 0.87, RMSEA = 0.08, SRMR = 0.12, AICc = 6,628) and the cat-ownership model (Figure 1B; CFI = 0.89, RMSEA = 0.07, SRMR = 0.11, AICc = 6,585). In both models, the direct effects of dog ownership (*β* = 0.01, *p* = 0.92) and cat ownership (*β* = 0.08, *p* = 0.46) on well-being were not significant. In the dog-ownership model, significant structural paths were observed from dog ownership to family interactions (*β* = 0.30, *p* < 0.01), from family interactions to non family interactions (*β* = 0.35, *p* = 0.01), and from self-esteem to well-being (*β* = 0.53, *p* < 0.01). In the cat-ownership model, significant paths emerged from family interactions to non family interactions (*β* = 0.32, *p* = 0.04) and from self-esteem to well-being (*β* = 0.53, *p* < 0.01).

#### 3.2.2 Effects of current dog and cat ownership among women

For women, both SEMs demonstrated good model fit (dog ownership: Figure 2A; CFI = 0.97, RMSEA = 0.04, SRMR = 0.07, AICc = 7,408; cat ownership: Figure 2B; CFI = 0.99, RMSEA = 0.02, SRMR = 0.07, AICc = 7,334). As in men, the direct effects of dog ownership (*β* = 0.03, *p* = 0.74) and cat ownership (*β* = −0.02, *p* = 0.76) on well-being were not significant. In the dog-ownership model, significant structural paths were observed from dog ownership to family interactions (*β* = 0.19, *p* = 0.04) and non family interactions (*β* = 0.40, *p* < 0.01). Family interactions were significantly associated with non family interactions (*β* = 0.36, *p* < 0.01), general trust (*β* = −0.24, *p* = 0.04), and self-esteem (*β* = −0.28, *p* = 0.03). Non family interactions, in turn, were significantly associated with general trust (*β* = 0.38, *p* < 0.01), CEI (*β* = −0.31, *p* = 0.02) and self-esteem (*β* = 0.48, *p* < 0.01). Self-esteem was positively associated with well-being (*β* = 0.49, *p* < 0.01). In the cat-ownership model for women, significant paths emerged from family interactions to non family interactions (*β* = 0.51, *p* < 0.01), general trust (*β* = −0.33, *p* = 0.02), CEI (*β* = 0.29, *p* = 0.03), and self-esteem (*β* = −0.38, *p* = 0.02). In addition, non family interactions were significantly associated with general trust (*β* = 0.52, *p* < 0.01), CEI (*β* = −0.39, *p* < 0.01), and self-esteem (*β* = 0.60, *p* < 0.01). Self-esteem was positively associated with well-being (*β* = 0.49, *p* < 0.01).

#### 3.2.3 Effects of attachment to pets and interaction with pets

Additional SEM analyses examined whether pet interaction patterns and attachment to pets were associated with well-being through social relationships. Overall, physical contact time with pets was not significantly associated with well-being, either directly or indirectly. In contrast, greater pet caregiving time was associated with higher levels of family and non family interactions, which were in turn linked to well-being through social and psychological variables.

Basic attachment to pets showed both a marginal direct association with well-being and an indirect association through non family interactions. Similarly, close attachment to pets was indirectly associated with well-being through family and non family interactions. These findings suggest that the quality of human–pet relationships, particularly caregiving involvement and emotional attachment, may be linked to well-being through social pathways. Detailed model fit indices and parameter estimates are provided in Supplementary Figures S4–S7.

### 3.3 Study 2: exploring physiological mechanisms

#### 3.3.1 Associations with hormones

Results of the generalized linear mixed model analyses indicated that neither dog ownership (*β* = 0.053, *p* = 0.207), cat ownership (*β* = 0.055, *p* = 0.309), age (*β* = 0.002, *p* = 0.266), nor gender (*β* = −0.007, *p* = 0.866) was significantly associated with urinary cortisol concentration. Partial correlation analyses were conducted to examine the associations between urinary cortisol concentrations and each variable, controlling for age and sex. The results indicated that urinary cortisol concentrations were significantly negatively correlated with the amount of conversation with a partner (*r* = −0.447, *p* = 0.013; Supplementary Table S5).

#### 3.3.2 Associations with the gut microbiota

**Figure 3:**
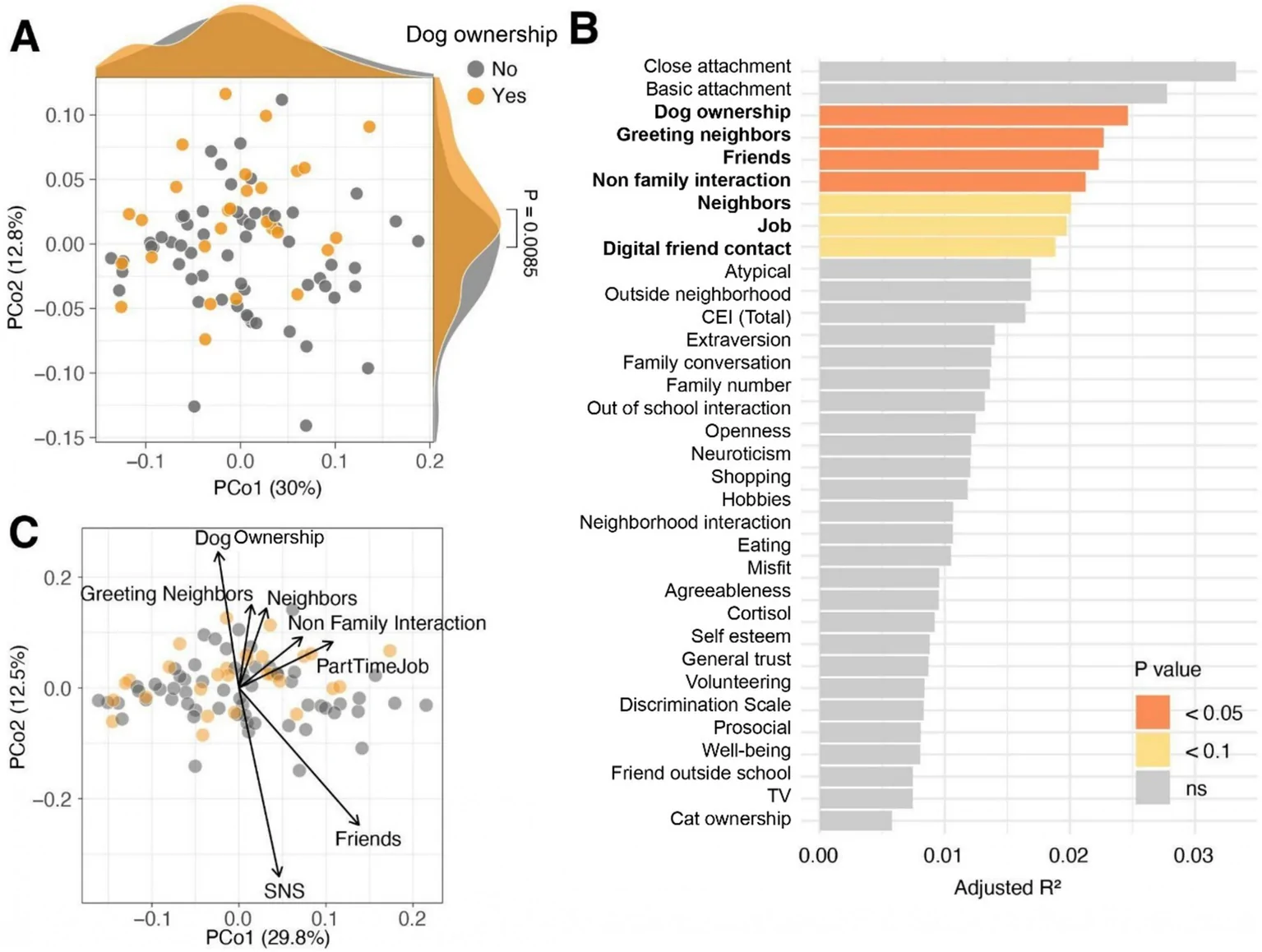
Associations between gut microbiota composition, dog and cat ownership, interactions with others, and psychosocial factors. **(A)** Principal coordinates analysis (PCoA) based on weighted UniFrac distances showing the overall gut microbial community structure according to dog ownership. Statistical significance was assessed using permutational multivariate analysis of variance (PERMANOVA). **(B)** Distance-based redundancy analysis (db-RDA) illustrating the associations between gut microbiota composition and explanatory variables. Variables shown significantly explained variation in microbial community structure. **(C)** Envfit analysis showing the direction and strength of associations between explanatory variables and gut microbiota composition in the ordination space. Arrows indicate the direction of increasing values for each variable, and arrow length reflects the strength of the association.

Principal coordinates analysis (PCoA) based on weighted UniFrac distances indicated that the overall structure of the gut microbiota tended to differ partially according to dog ownership. Permutational multivariate analysis of variance (PERMANOVA) revealed that the effect of dog ownership was statistically significant (Figure 3A; *R*^2^ = 0.026, *p* = 0.024), suggesting that although modest in magnitude, dog ownership accounted for a small but significant proportion of variance in gut microbial community structure. Furthermore, distance-based redundancy analysis (db-RDA) identified several factors that significantly contributed to variation in gut microbiota composition, including dog ownership (*R*^2^ = 0.025, *p* = 0.024), out of school interaction (*R*^2^ = 0.022, *p* = 0.036), greeting neighbors (*R*^2^ = 0.023, *p* = 0.038), and non family interactions (*R*^2^ = 0.021, *p* = 0.044). In addition, marginal trends were observed for neighborhood interaction (*R*^2^ = 0.020, *p* = 0.058), jobs (*R*^2^ = 0.020, *p* = 0.067), and interactions via social networking services (DFC; *R*^2^ = 0.019, *p* = 0.087) (Figure 3B). Results from the envfit analysis indicated that dog ownership and non family interactions (e.g., greeting neighbors) explained microbial variation along a similar directional gradient, whereas DFC were oriented in the opposite direction relative to dog ownership (Figure 3C).

## 4 Discussion

The present study addressed three questions regarding the pathways linking companion animals to human well-being. First, we examined whether dog and cat ownership were associated with closer social relationships and whether these relationships were linked to well-being. Second, we evaluated the relative importance of direct and indirect pathways from companion animals to well-being. Third, we explored whether physiological factors, including cortisol concentrations and gut microbiota composition, were associated with these pathways. Overall, the findings suggest that companion animals contribute to well-being primarily through indirect social pathways rather than through direct effects. Dog ownership was associated with family and non family interactions, particularly among women, whereas comparable effects were not consistently observed for cat ownership. In addition, both cortisol concentrations and gut microbiota composition were associated with social interaction patterns, suggesting that biological factors may be involved in these processes.

The first aim of this study was to examine whether dog and cat ownership were associated with social relationships in everyday life. Consistent with our hypotheses, dog ownership was associated with family and non family interactions. Interestingly, the structural model among women revealed two seemingly contrasting pathways. While dog ownership increased family interaction, the pathway mediated solely through family interaction was associated with lower self-esteem and well-being. In contrast, the pathway in which family interaction extended to interactions with the local community and friends was associated with higher self-esteem and well-being. These findings suggest that family interaction itself does not necessarily enhance well-being; rather, its psychological benefits may depend on whether it extends beyond the household to broader social connections. Dogs are well known to function as social catalysts, facilitating interactions with neighbors and other community members through activities such as walking (McNicholas and Collis, 2000; Wood et al., 2005; Wood et al., 2015). When social relationships remain centered within the family, social resources may be confined to the household. By contrast, when dogs facilitate connections with the wider community, individuals may gain greater social support, opportunities to develop meaningful social roles, and a stronger sense of belonging, which may ultimately enhance self-esteem and well-being. In contrast, cats are generally less likely to create opportunities for interactions outside the household, which may explain the weaker associations observed for cat ownership. These findings suggest that companion animals influence human social relationships in species-specific ways, with dogs showing a stronger association with both family and non family interactions.

The second aim of this study was to evaluate the relative importance of direct and indirect pathways linking companion animals to well-being. Across the SEMs, direct effects of dog or cat ownership on well-being were generally not observed. Instead, companion animals were associated with well-being primarily through indirect pathways involving family and non family interactions. These findings suggest that the benefits of companion animals may not arise simply from ownership itself, but rather from the ways in which companion animals shape everyday social experiences. Family and community relationships were consistently associated with self-esteem, trust, and well-being, indicating that social relationships may represent an important mechanism through which companion animals contribute to psychological well-being.

The absence of robust direct effects may also help explain the inconsistent findings reported in previous studies of pet ownership and mental health. If the effects of companion animals depend on whether ownership promotes meaningful social relationships, the benefits of ownership are likely to vary according to individual circumstances and social environments. The present findings therefore support the view that companion animals contribute to well-being primarily as facilitators of social relationships rather than as direct sources of psychological benefit.

The present study provided the results that the pathways linking companion animals and well-being would differ by gender. Indirect pathways involving family and non family interactions were more clearly observed among women than among men. Previous studies have reported that women tend to show stronger emotional attachment to companion animals and greater involvement in daily caregiving activities (Herzog, 2011; Power, 2008). Such involvement may increase opportunities for social interactions within both family and community settings, thereby strengthening the indirect pathways linking companion animals and well-being. However, the mechanisms underlying these gender differences remain unclear and warrant further investigation.

The third aim of this study was to explore potential physiological factors underlying the associations among companion animals, social relationships, and well-being. Although exploration, both cortisol concentrations and gut microbiota composition were associated with aspects of social interaction. Previous studies have reported differences in gut microbiota composition between dog owners and nonowners (Song et al., 2013; Tun et al., 2017). Furthermore, Miyauchi et al. (2025) suggested that gut microbiota of dog ownership influences social behavior. Together, these findings suggest that physiological factors may represent potential biological correlates of the social pathways identified in this study. However, further research is needed to clarify the underlying mechanisms.

Several limitations of this study should be acknowledged. First, the sample size was limited, particularly with respect to cat owners, which restricts the robustness of conclusions regarding cat ownership effects. Second, mental health indicators were assessed at a single time point, limiting causal inference due to the cross-sectional design. In addition, potentially important confounding variables, such as income level and personality traits, could not be controlled for and should be considered when interpreting the findings. These factors may represent potential confounders because they may influence both pet ownership status and well-being outcomes. Future studies incorporating larger samples, longitudinal designs, and family-unit level including data collected from all family members may provide a more comprehensive understanding of how pet ownership influences mental health.

Taken together, the present findings support a framework in which companion animals contribute to human well-being through interconnected social and biological pathways. Rather than acting as direct determinants of well-being, companion animals appear to influence everyday social experiences, particularly within family and community contexts, which are themselves associated with psychological well-being. In addition, the associations observed with cortisol concentrations and gut microbiota composition suggest that physiological processes may be linked to these social pathways. These findings highlight the importance of considering companion animals not only as sources of emotional support but also as factors that shape broader social and biological environments.

## Data Availability

The raw data supporting the conclusions of this article are available from the corresponding author upon reasonable request.

## Footnotes

1
https://www.jikken-baito.com
