# Supplementary material for: Social and biological pathways linking relationships with companion animals to human well-being
Source: Front Psychol. 2026 Sep 3;17:1810355. doi: 10.3389/fpsyg.2026.1810355 (PMC13581753; doi:10.3389/fpsyg.2026.1810355)
Supplement: Supplementary file 1 [file Presentation_1.PDF]

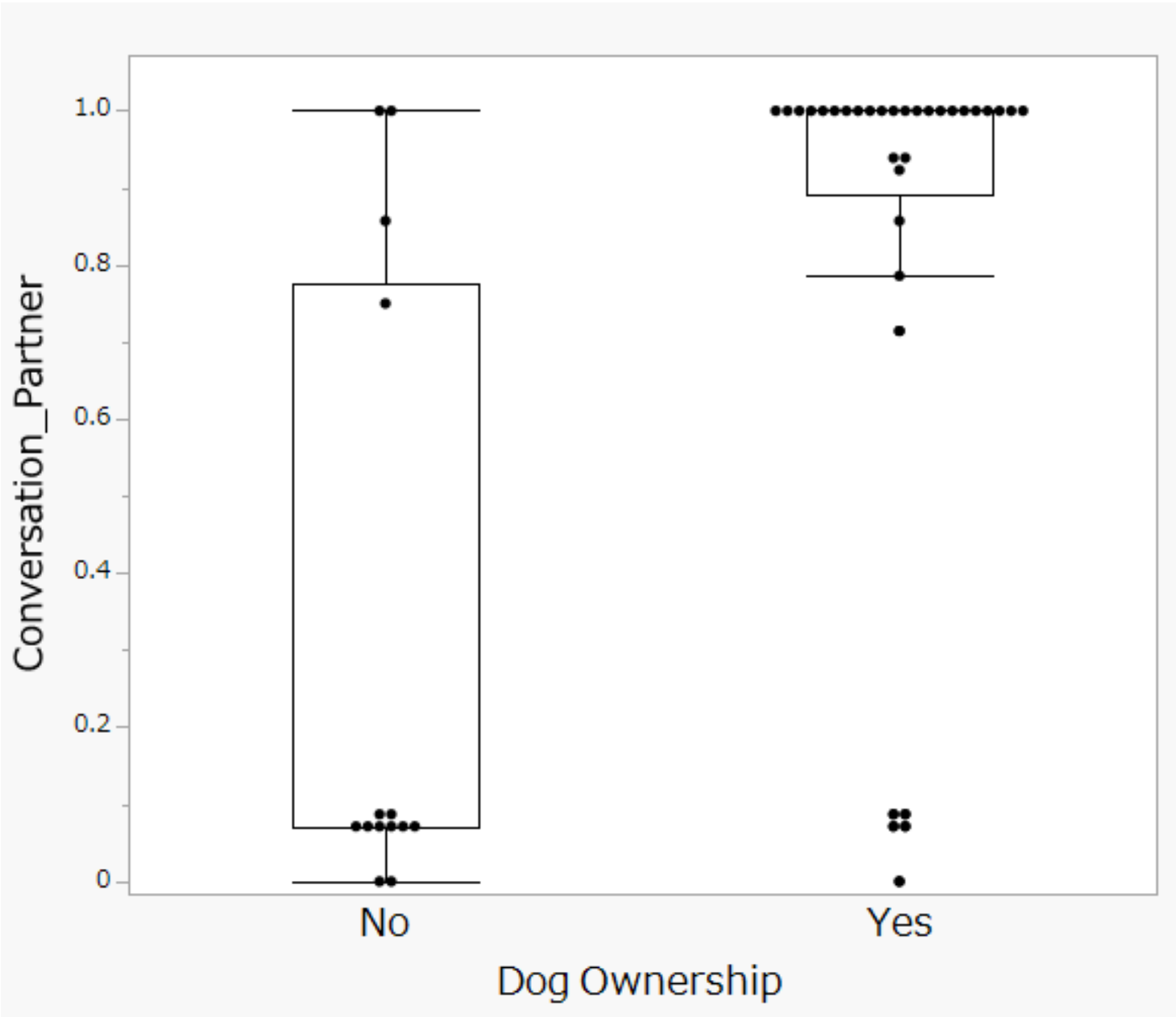


Figure S1A. Comparison of the proportion of conversations with partners between dog owners and non-dog owners. The vertical axis shows the proportion of days (out of two weeks) on which conversations with the partner occurred.

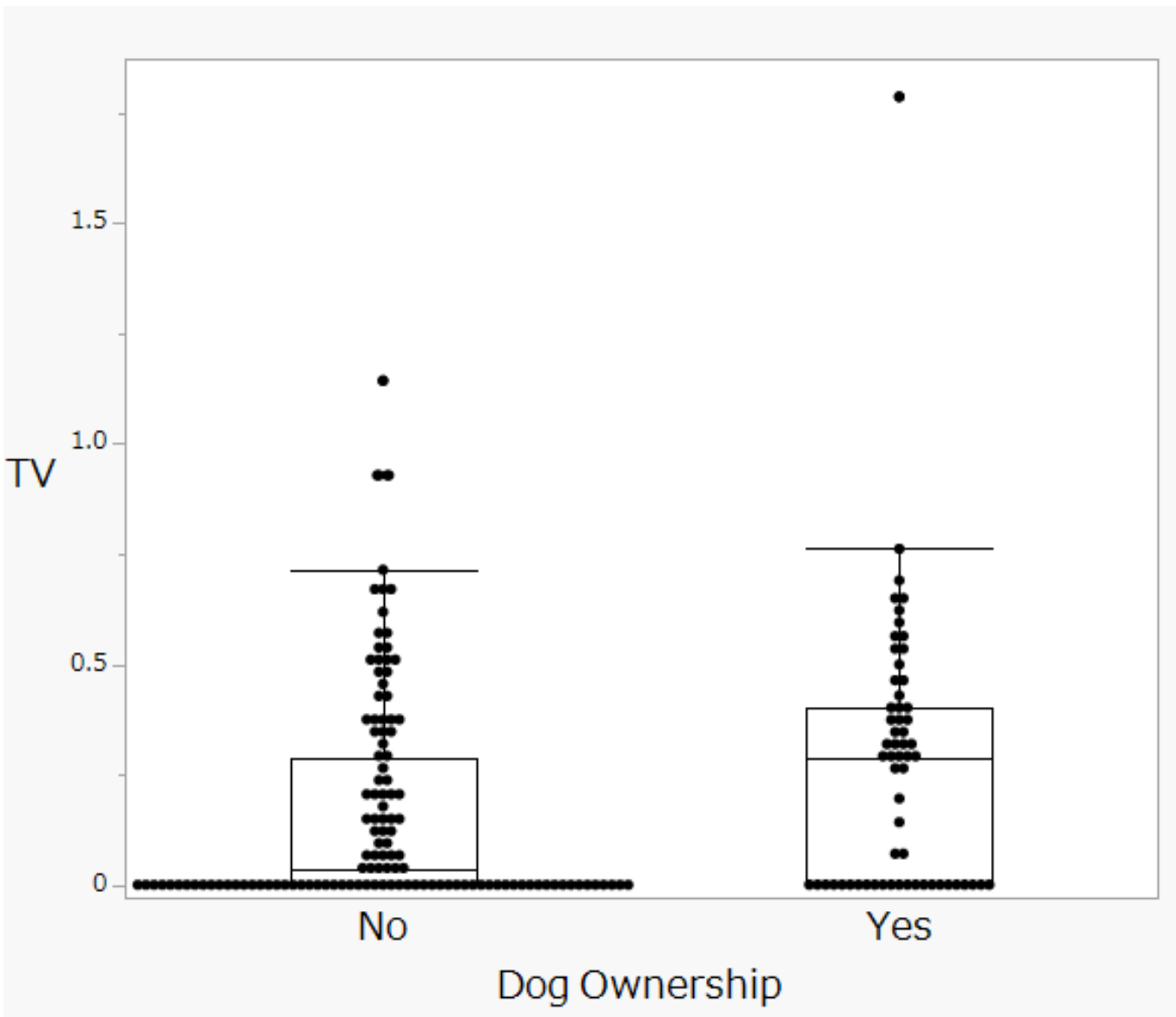


Figure S1B. Comparison of the proportion of watching TV with family members between dog owners and non-dog owners. The vertical axis shows the proportion of days (out of two weeks) on which watching TV with the family members (taking into account the number of people) occurred

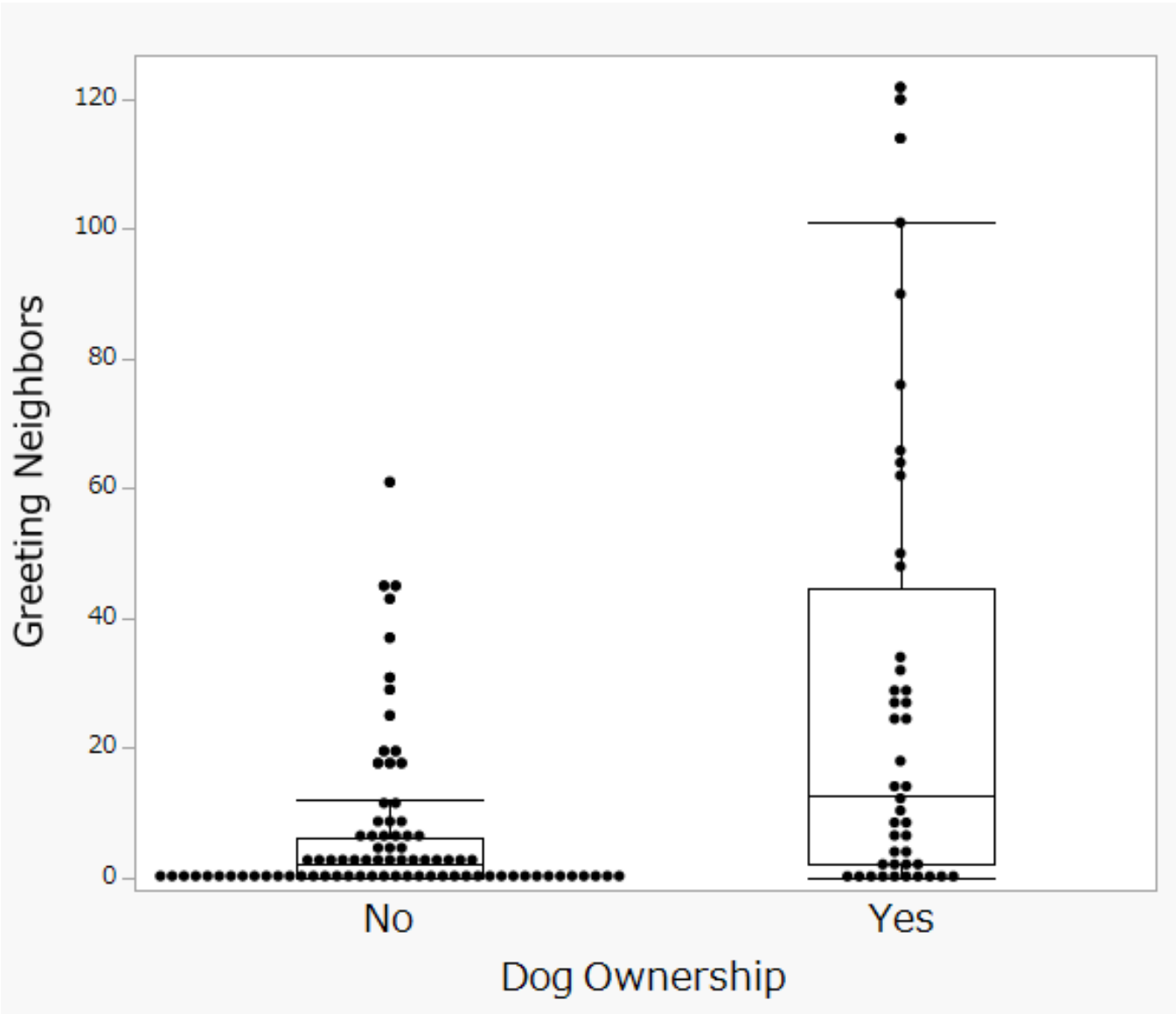


Figure S1C. Comparison of the proportion of greeting neighbors between dog owners and non-dog owners. The vertical axis shows the total number of people on which greeting neighbors occurred in two weeks.

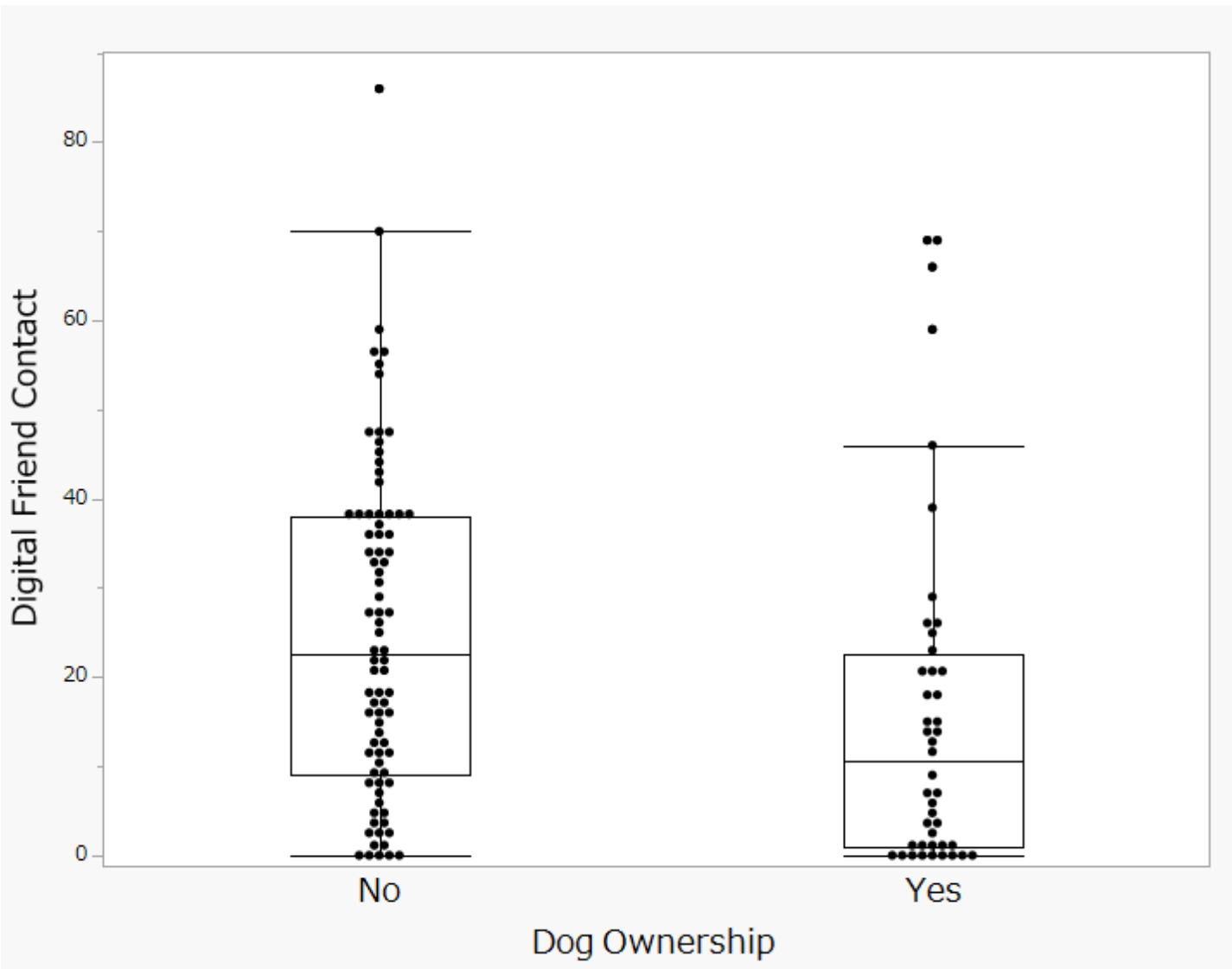


Figure S1D. Comparison of the proportion of interaction via SNS between dog owners and non-dog owners. The vertical axis shows the total number of people on which interaction via SNS occurred in two weeks.

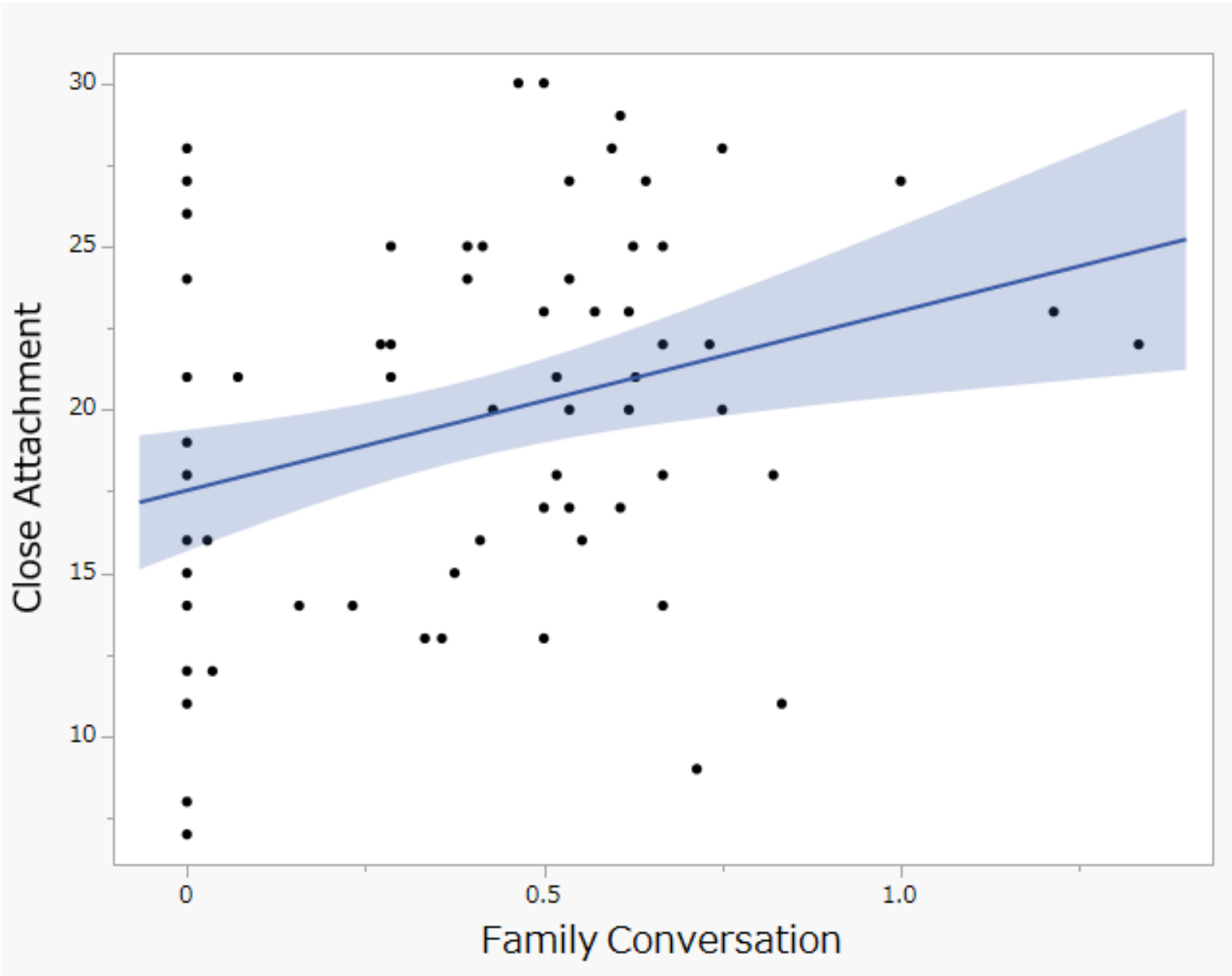

Figure S2A. Correlation between conversation with family members and close attachment to pets. The “family Conversation” shows the proportion of days (out of two weeks) on which conversations with family members (taking into account the number of people) occurred.

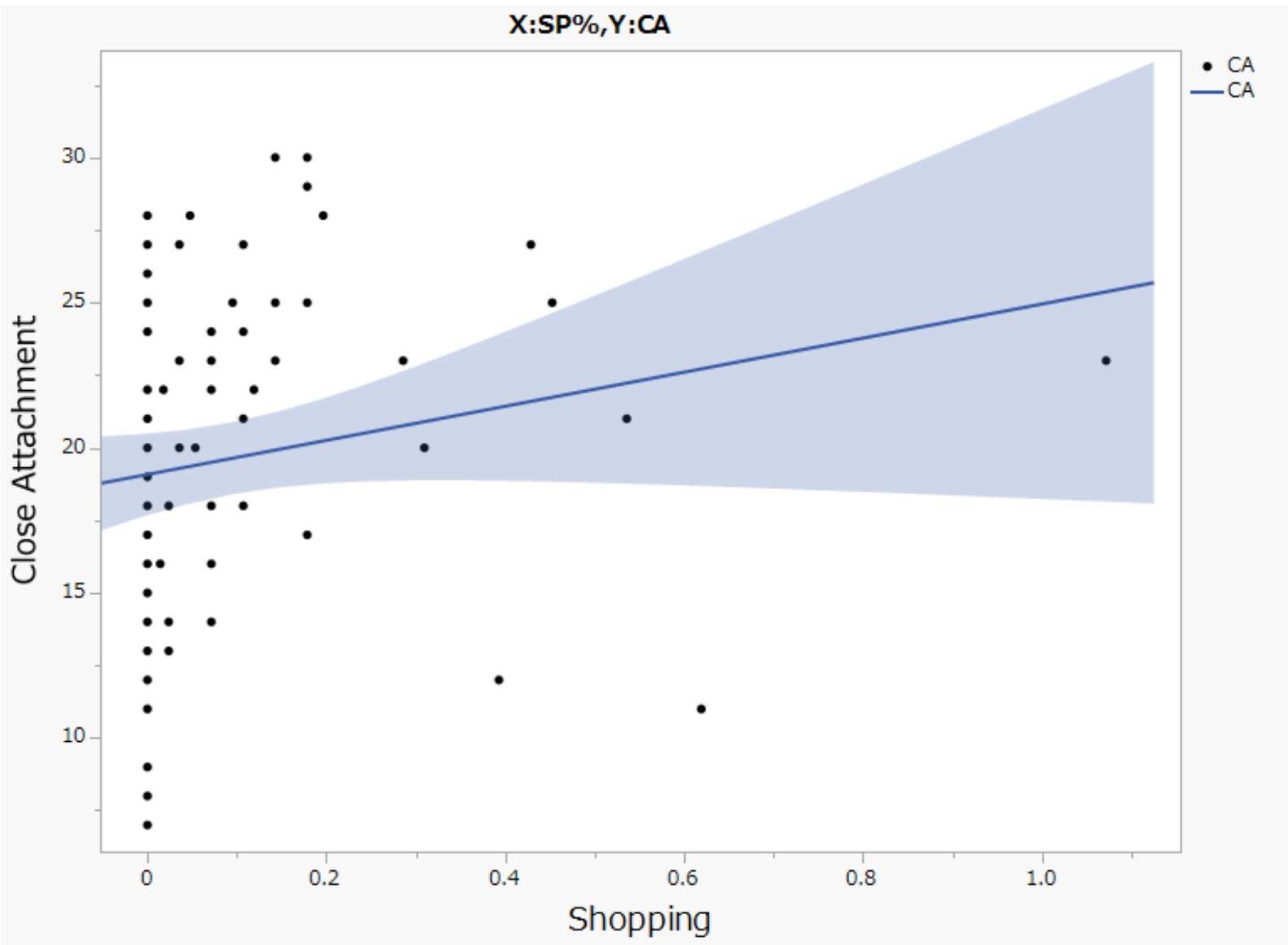

Figure S2B. Correlation between shopping with family members and close attachment to pets. The “Shopping” shows the proportion of days (out of two weeks) on which shopping with family members (taking into account the number of people) occurred.

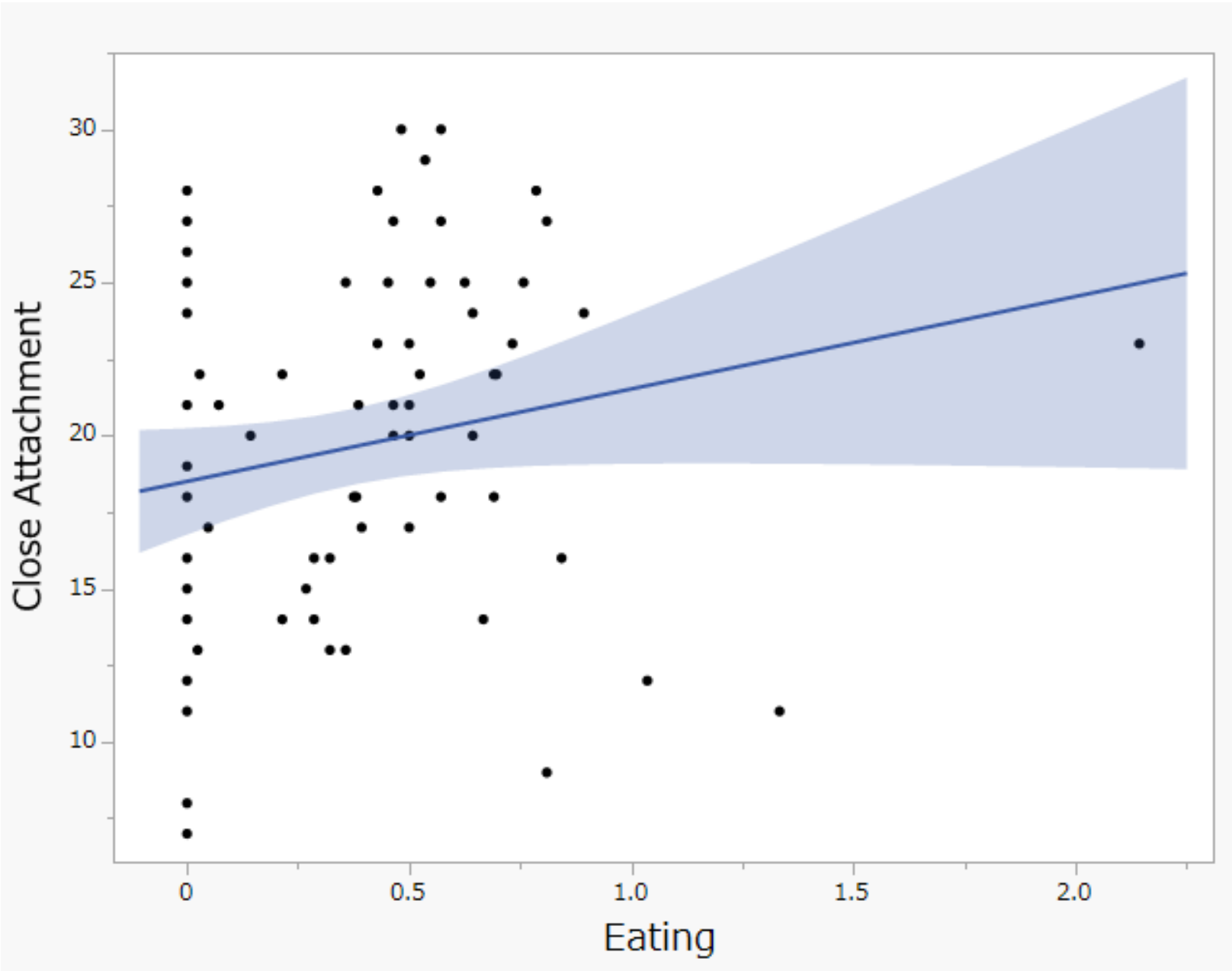


Figure S2C. Correlation between eating with family members and close attachment to pets. The “Eating” shows the proportion of days (out of two weeks) on which eating with family members (taking into account the number of people) occurred.

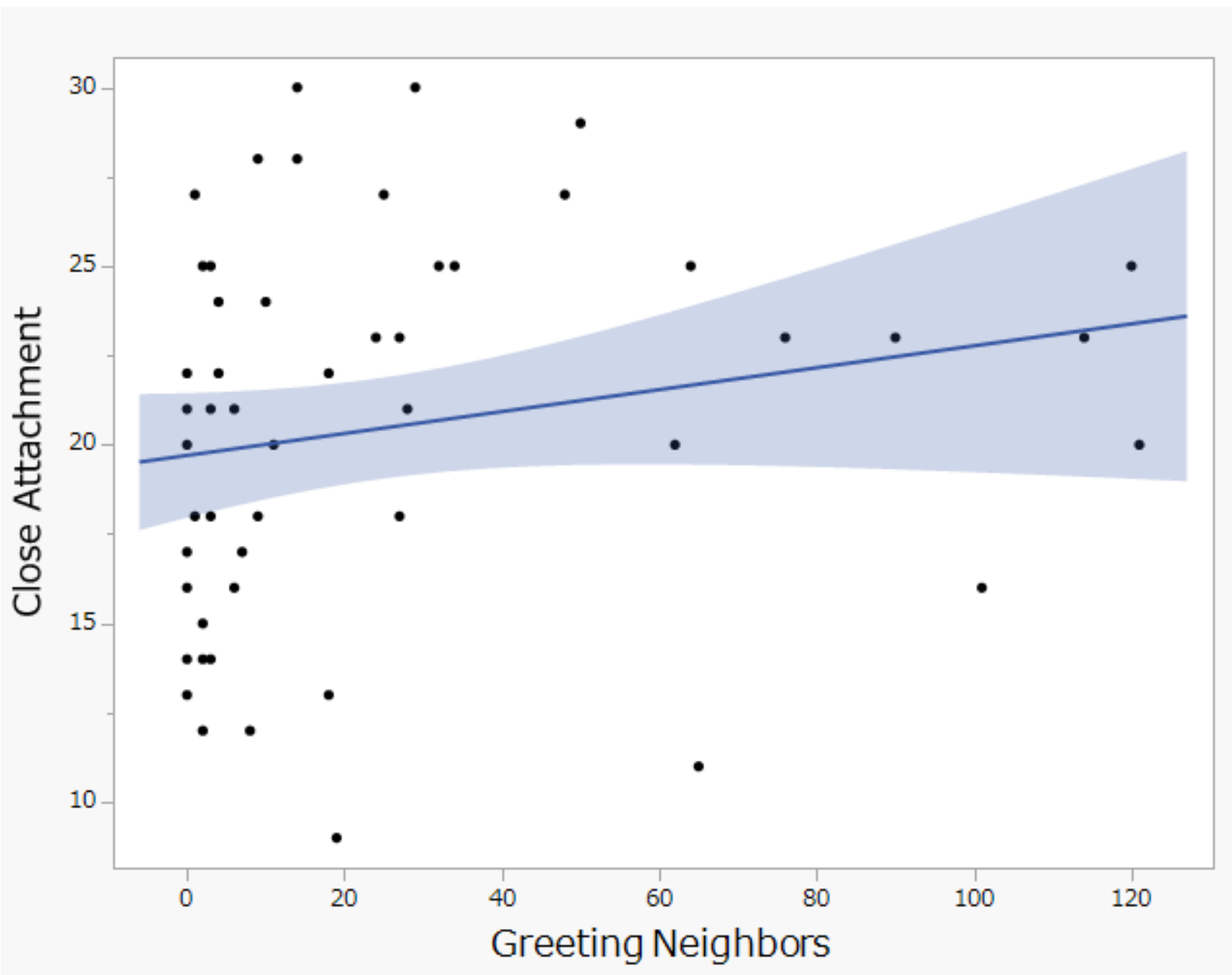


Figure S2D. Correlation between greeting neighbors and close attachment to pets. The "Greeting Neighbors” axis shows the total number of people on which greeting neighbors occurred in two weeks.

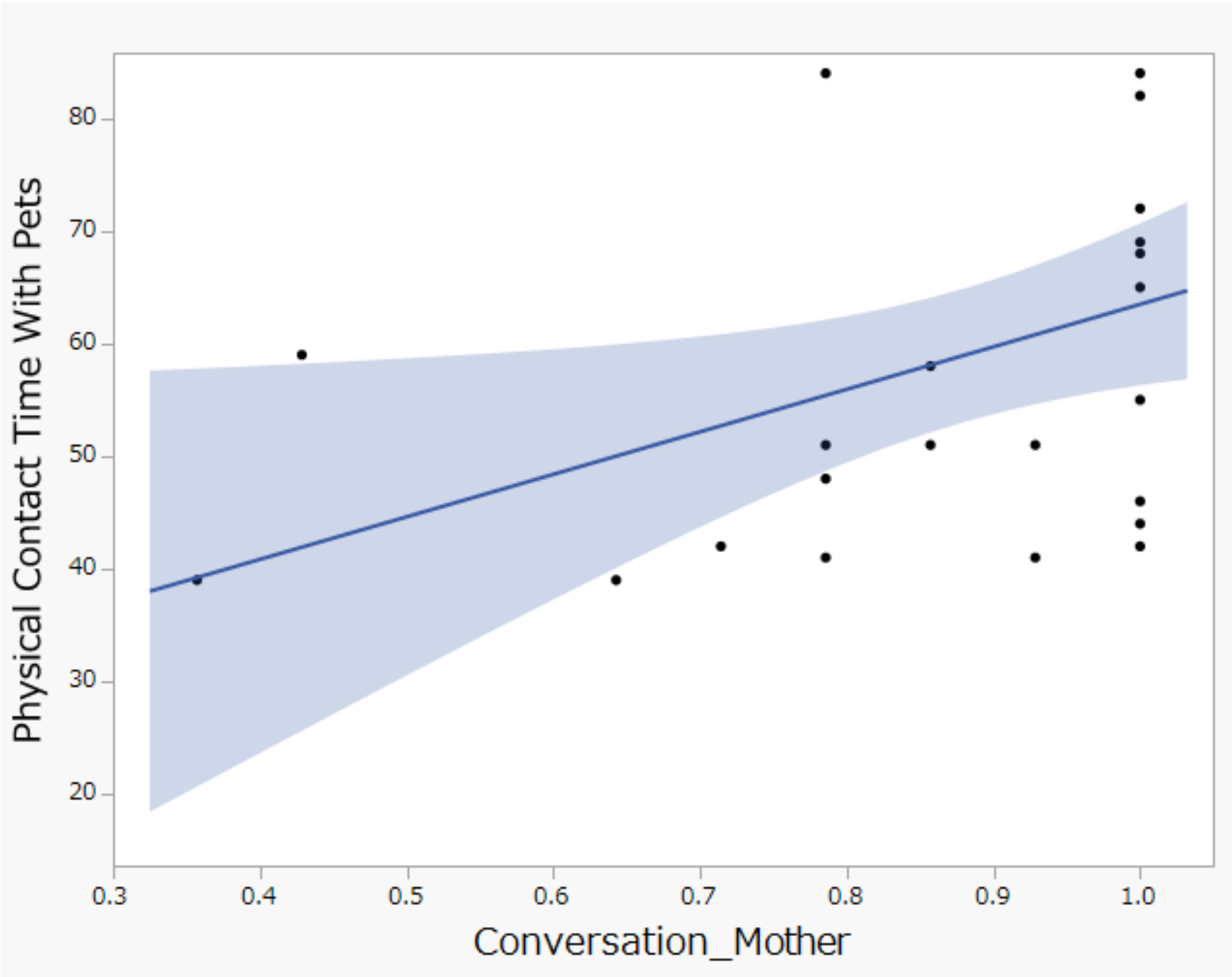


Figure S2E. Correlation between conversation with mother and physical contact with pets. The "Physical Contact Time With Pets" axis shows the total minute of physical contact with pets in two weeks. The "conversation_mother" shows the proportion of days (out of two weeks) on which conversations with mother occurred.

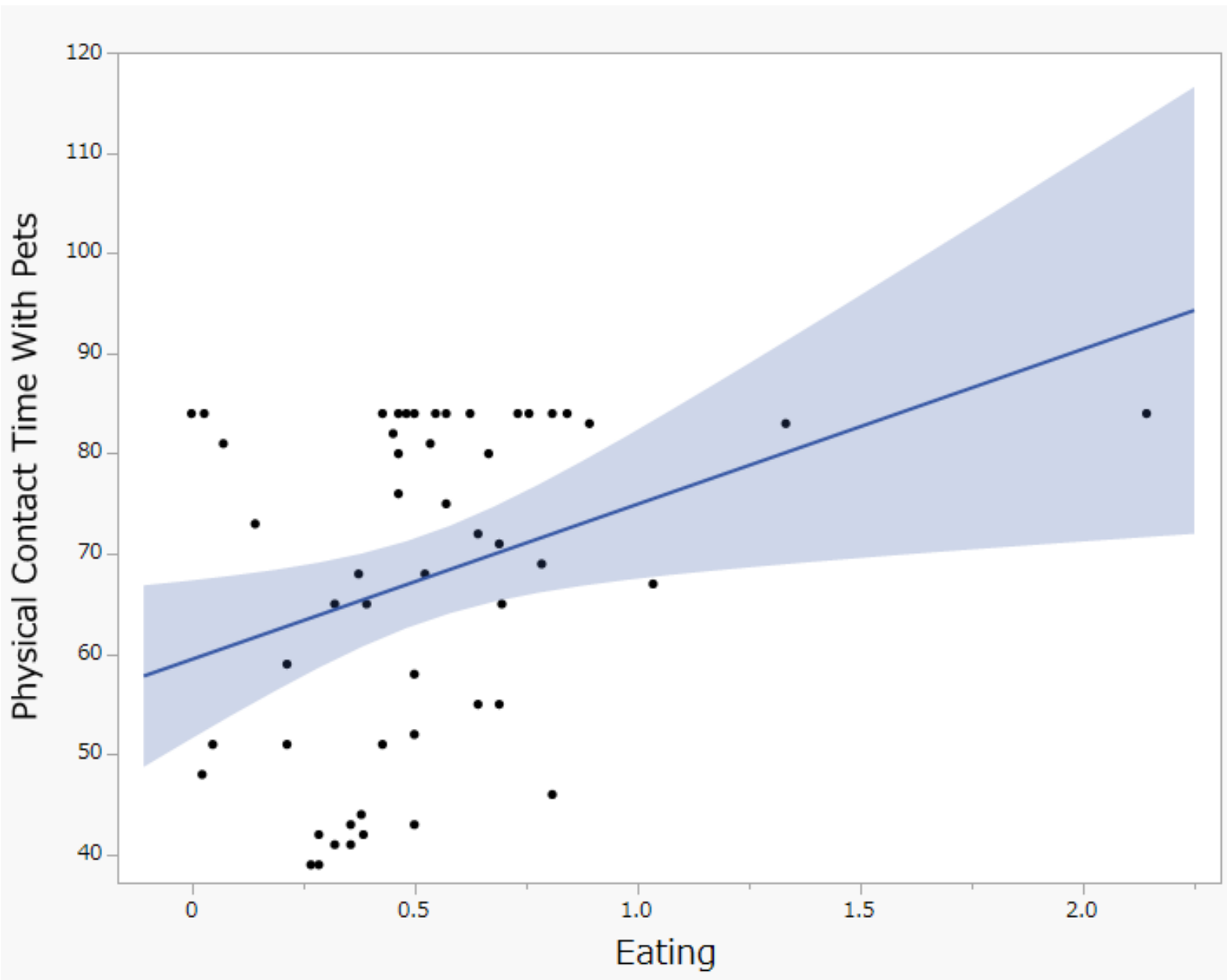


Figure S2F. Correlation between eating with family members and physical contact with pets. The "Physical Contact Time With Pets" axis shows the total minute of physical contact with pets in two weeks. The "eating" shows the proportion of days (out of two weeks) on which eating with family members (taking into account the number of people) occurred.

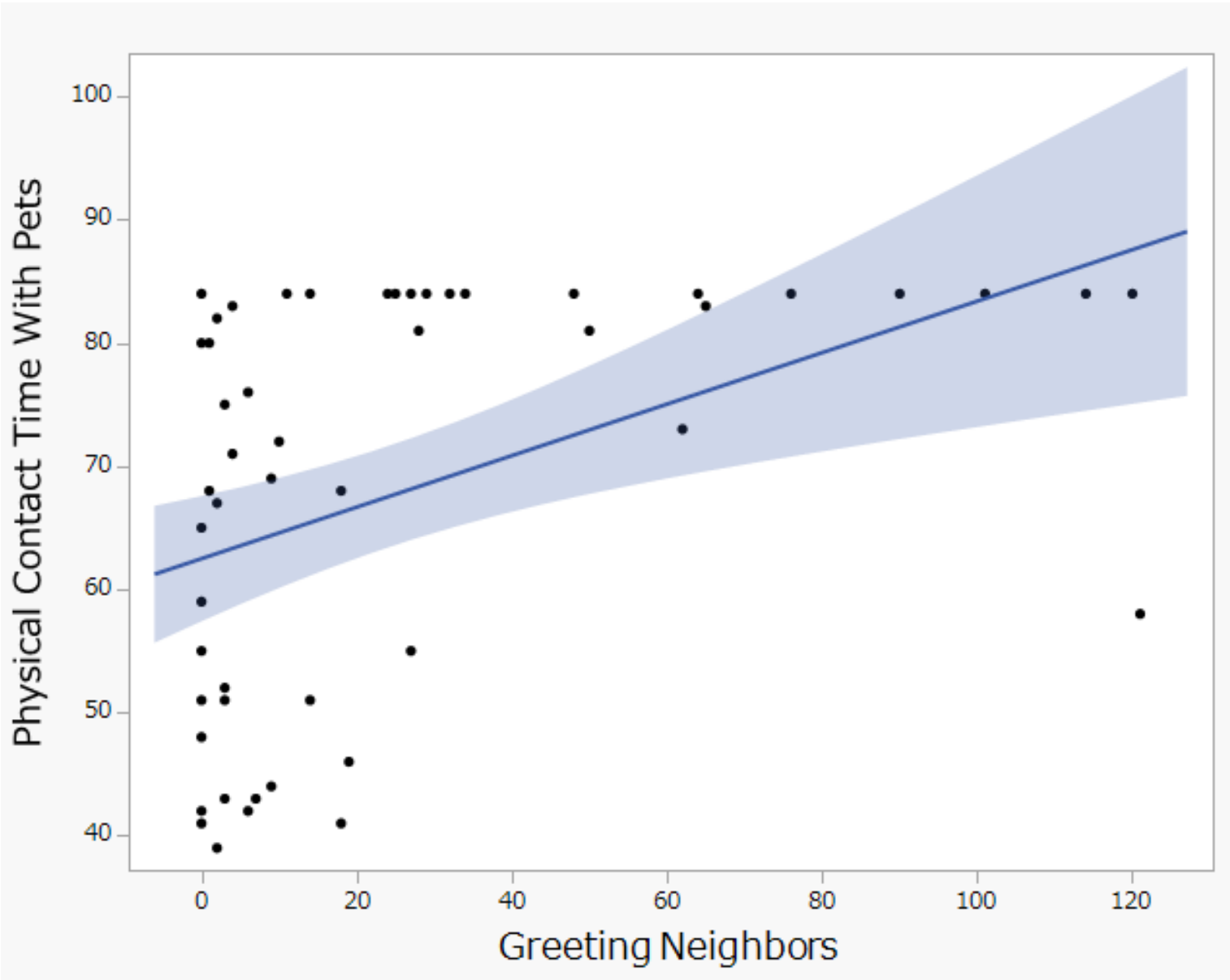


Figure S2G. Correlation between greeting neighbors and physical contact with pets. The "Physical Contact Time With Pets" axis shows the total minute of physical contact with pets in two weeks. The "Greeting Neighbors" axis shows the total number of people on which greeting neighbors occurred in two weeks.

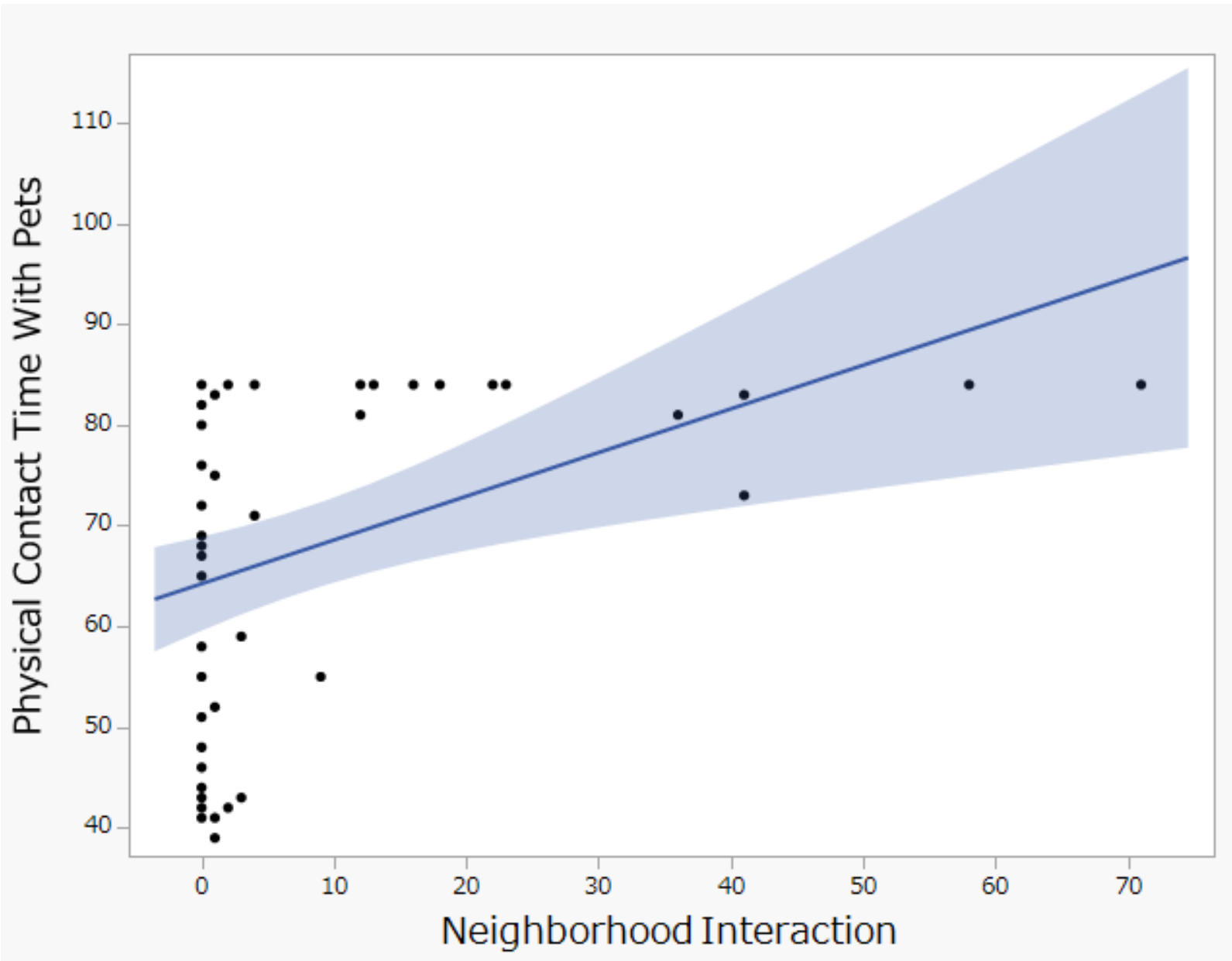


Figure S2H. Correlation between neighborhood interaction and physical contact with pets. The "Physical Contact Time With Pets" axis shows the total minute of physical contact with pets in two weeks. The "Neighborhood Interaction" axis shows the total number of people on which interaction with neighbors occurred in two weeks.

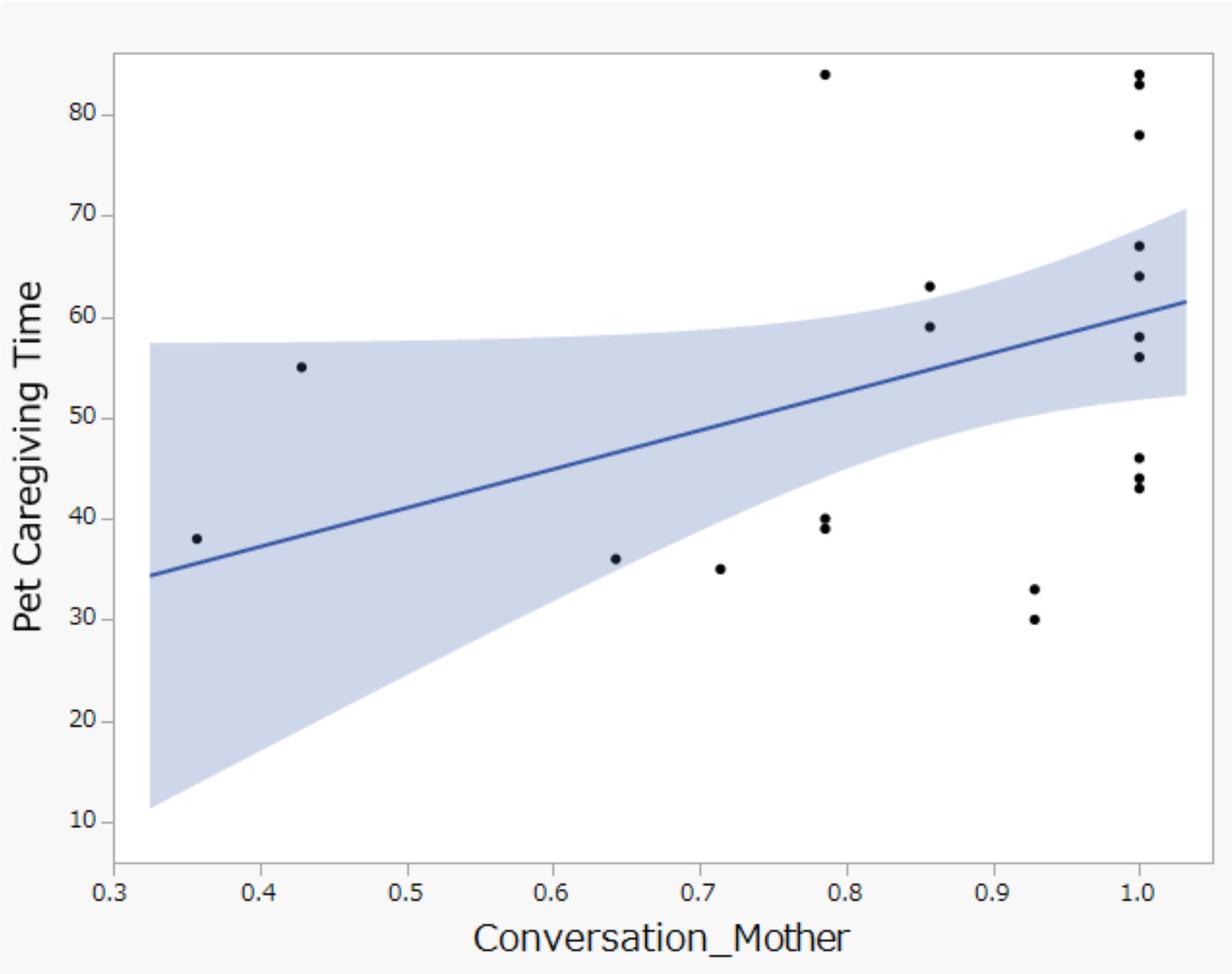


Figure S2I. Correlation between conversation with mother and pet caregiving. The "Pet Caregiving Time" axis shows the total minute of caregiving to pets in two weeks. The "conversation_mother" shows the proportion of days (out of two weeks) on which conversations with mother occurred.

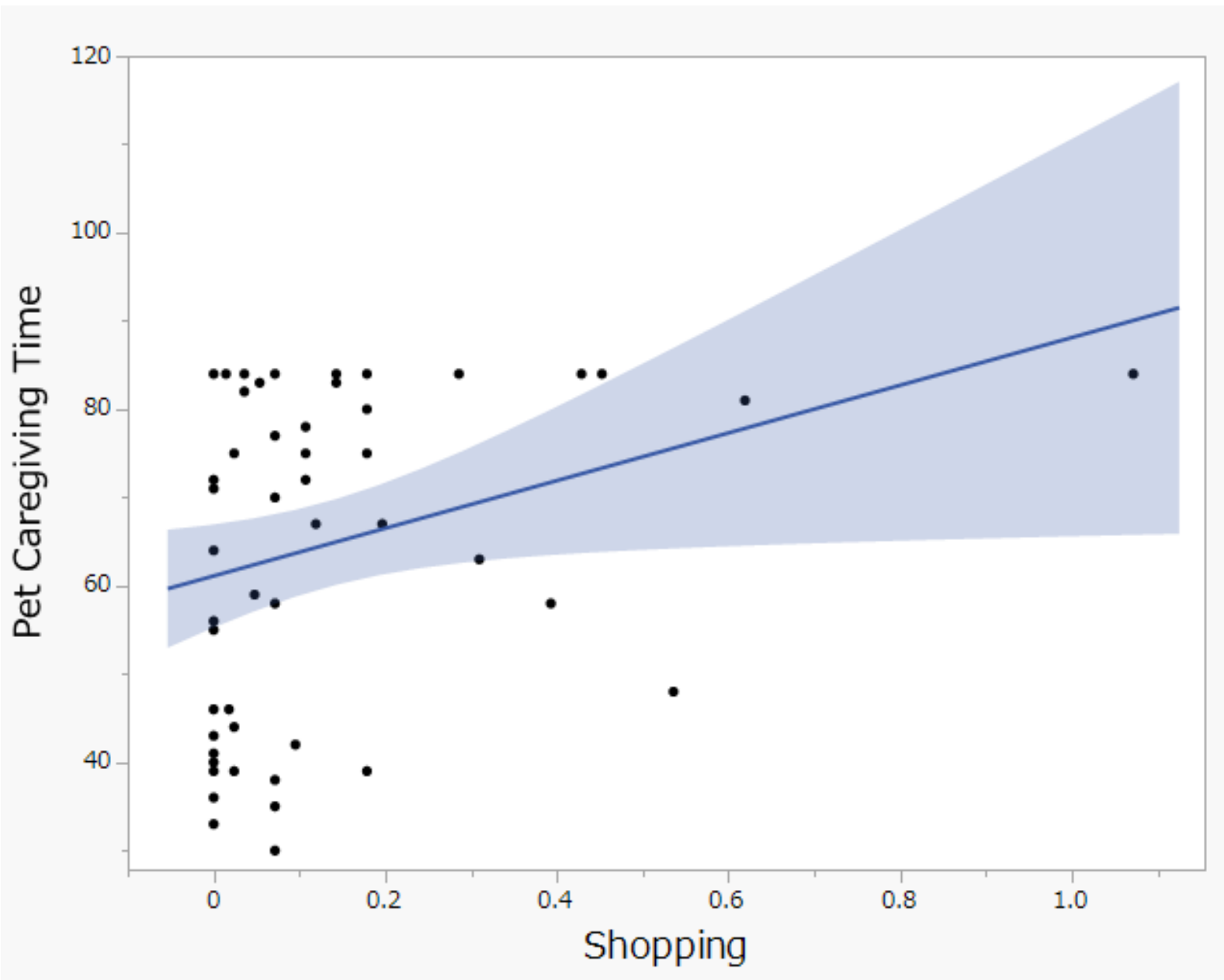


Figure S2J. Correlation between shopping with family members and pet caregiving. The "Pet Caregiving Time" axis shows the total minute of caregiving to pets in two weeks. The "Shopping" shows the proportion of days (out of two weeks) on which shopping with family members (taking into account the number of people) occurred.

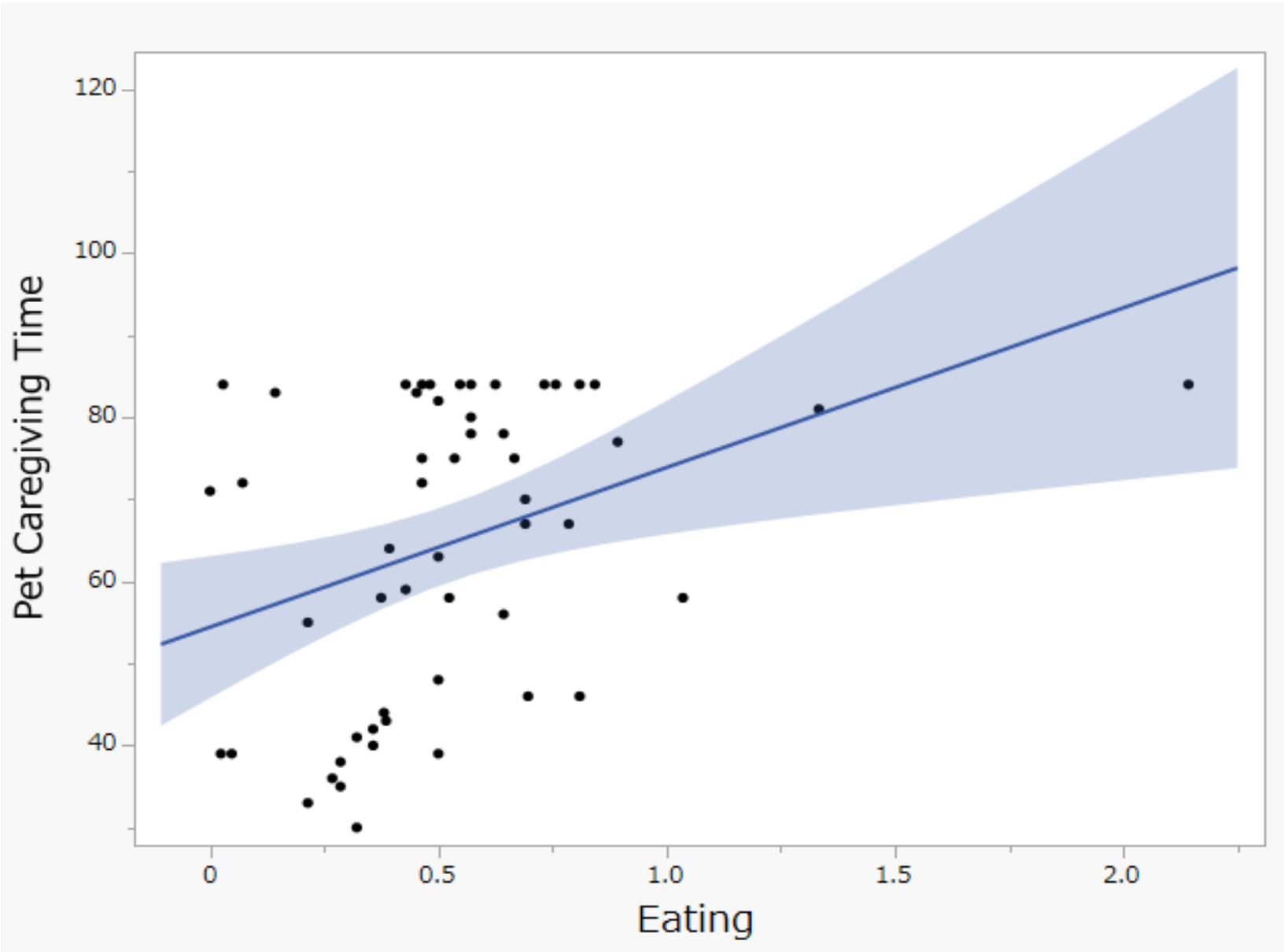

Figure S2K. Correlation between eating with family members and pet caregiving. The "Pet Caregiving Time” axis shows the total minute of caregiving to pets in two weeks. The “Eating” shows the proportion of days (out of two weeks) on which eating with family members (taking into account the number of people) occurred.

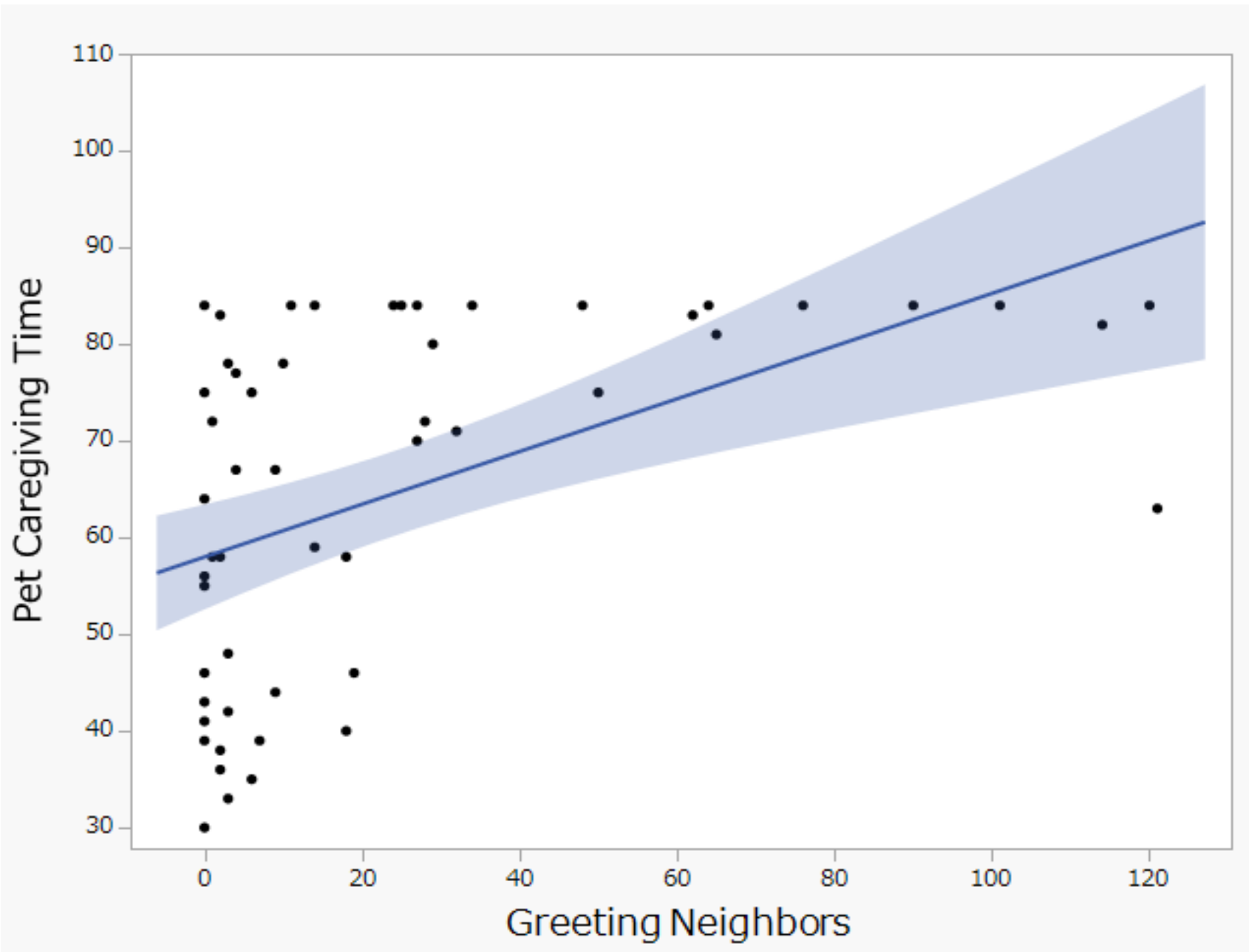

Figure S2L. Correlation between greeting neighbors and pet caregiving. The "Pet Caregiving Time” axis shows the total minute of caregiving to pets in two weeks. The "Greeting Neighbors” axis shows the total number of people on which greeting neighbors occurred in two weeks.

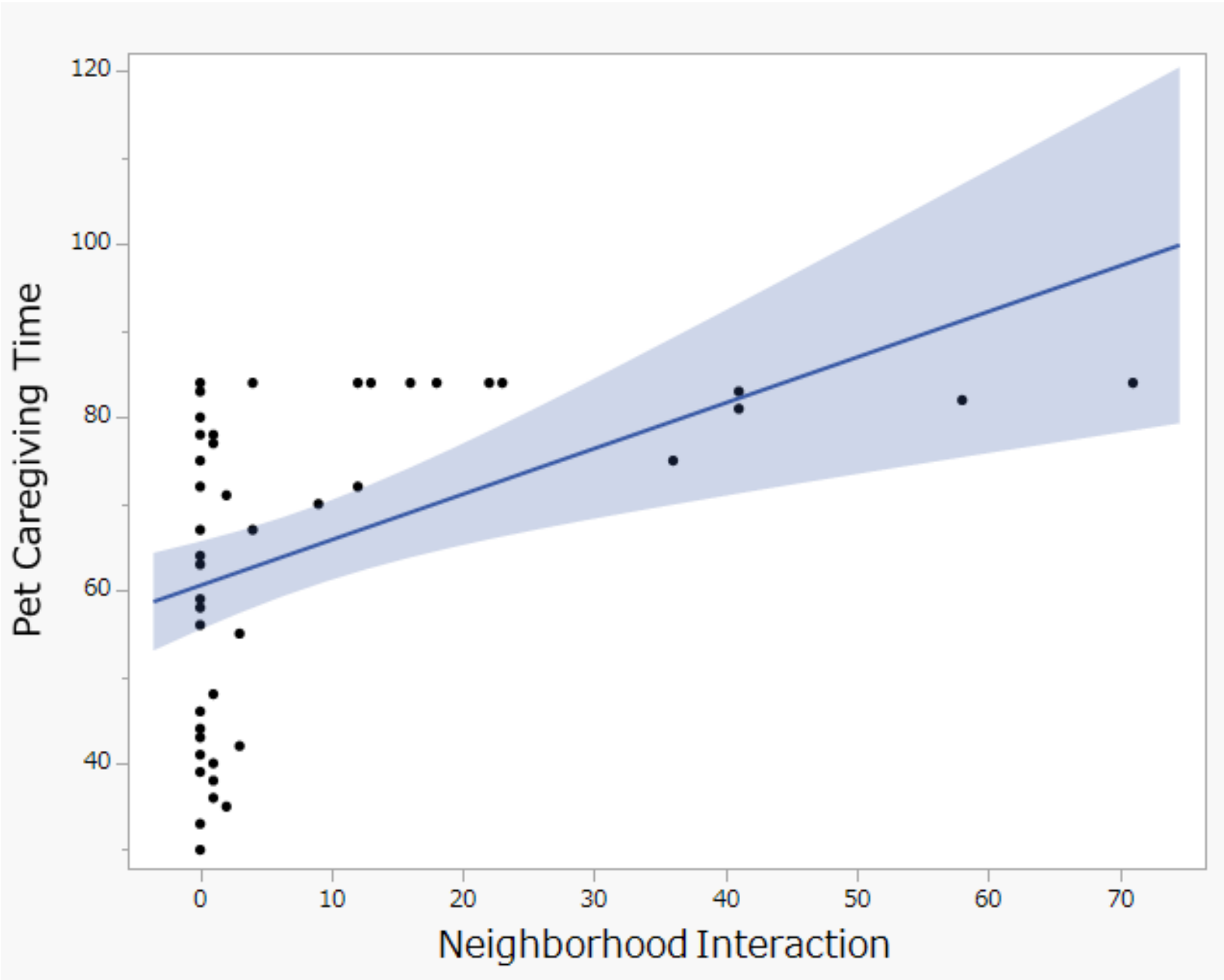


Figure S2M. Correlation between neighborhood interaction and pet caregiving. The "Pet Caregiving Time” axis shows the total minute of caregiving to pets in two weeks. The "Neighborhood Interaction” axis shows the total number of people on which interacting with neighbors occurred in two weeks.

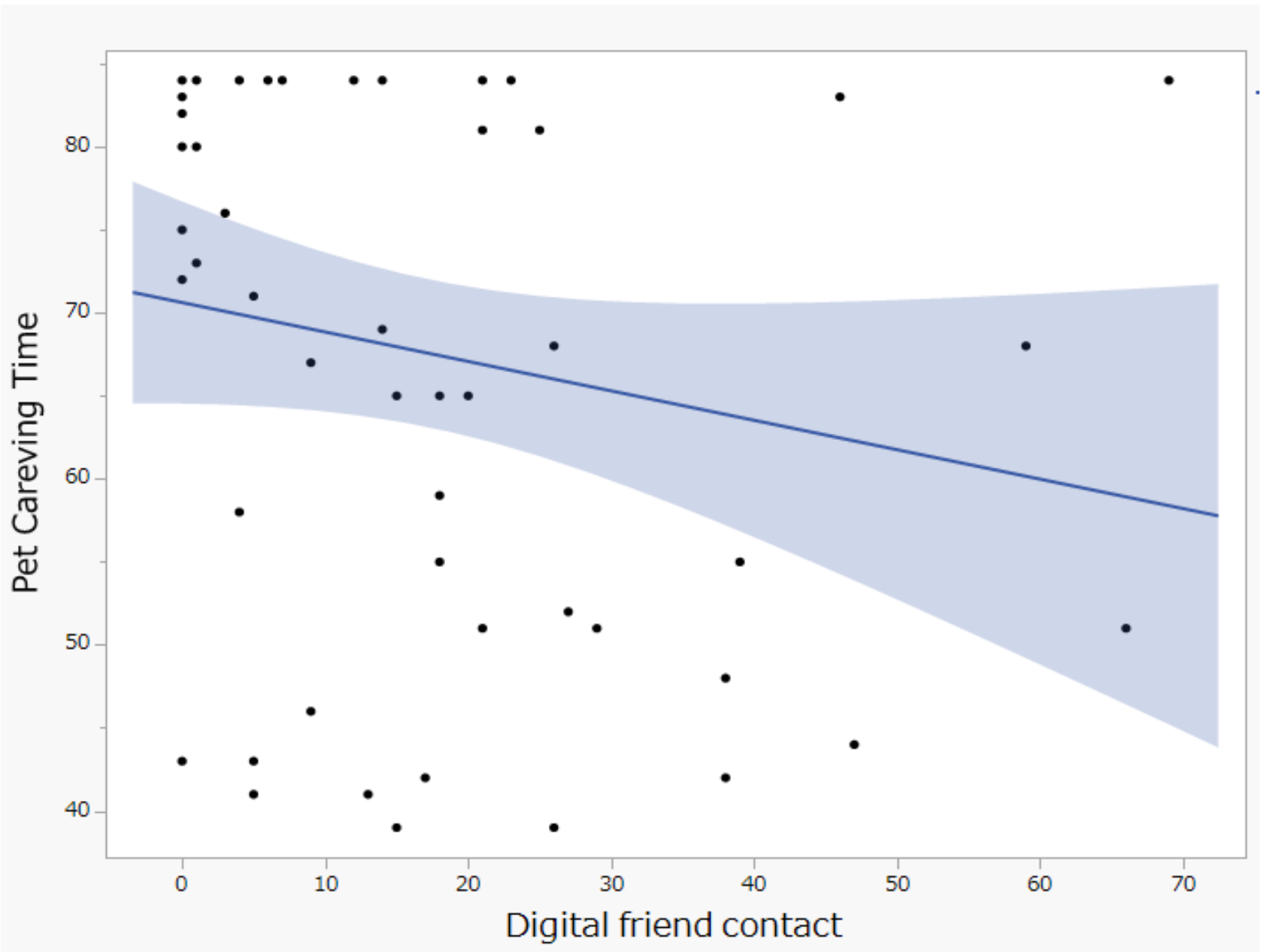


Figure S2N. Correlation between Digital friend contact and pet caregiving. The "Pet Caregiving Time” axis shows the total minute of caregiving to pets in two weeks. The "Digital friend contact” axis shows the total number of people on which contacting via computer or phone in two weeks.

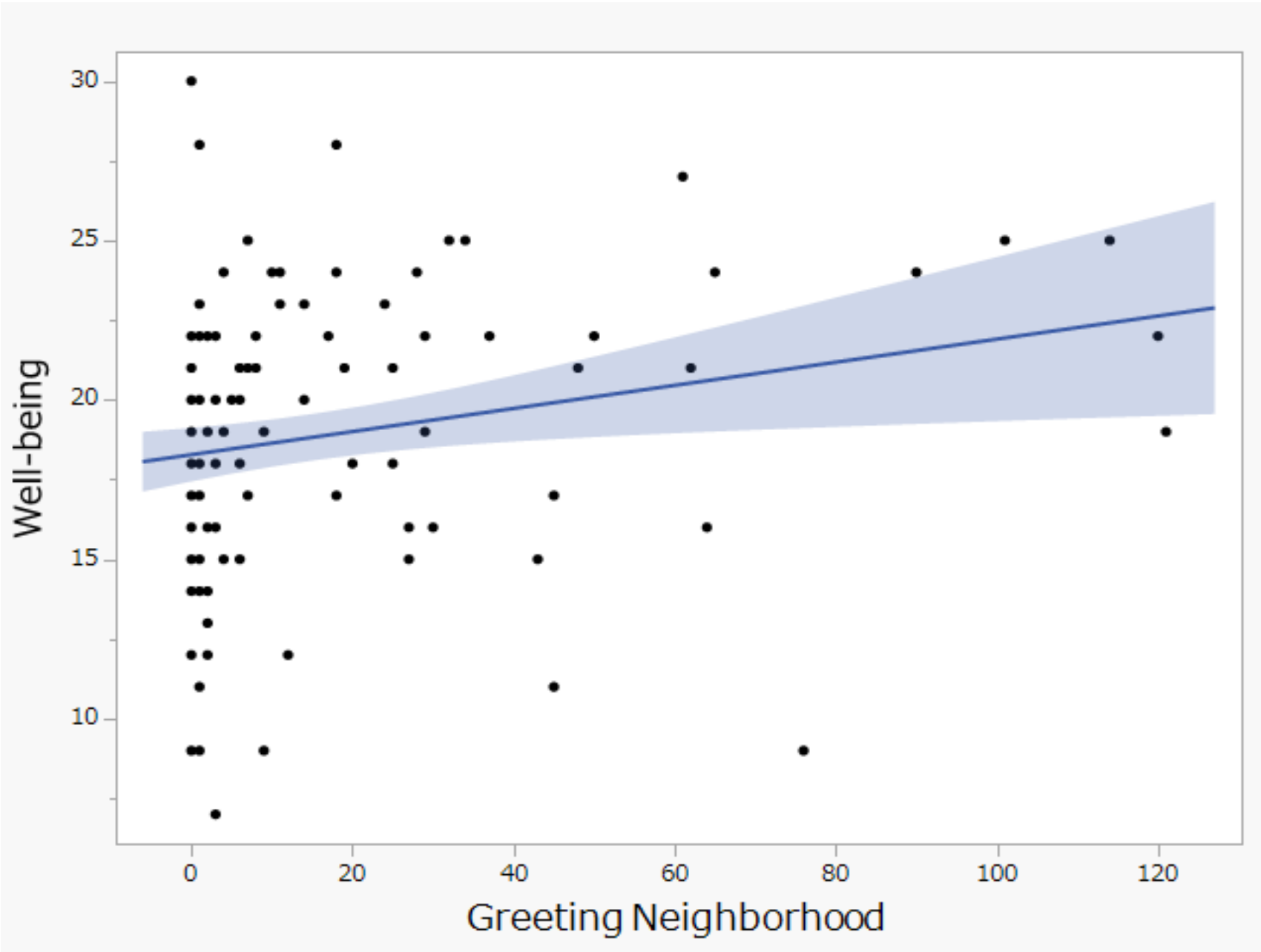


Figure S2O. Correlation between greeting neighbors and well-being. The "Greeting Neighbors" axis shows the total number of people on which greeting neighbors occurred in two weeks.

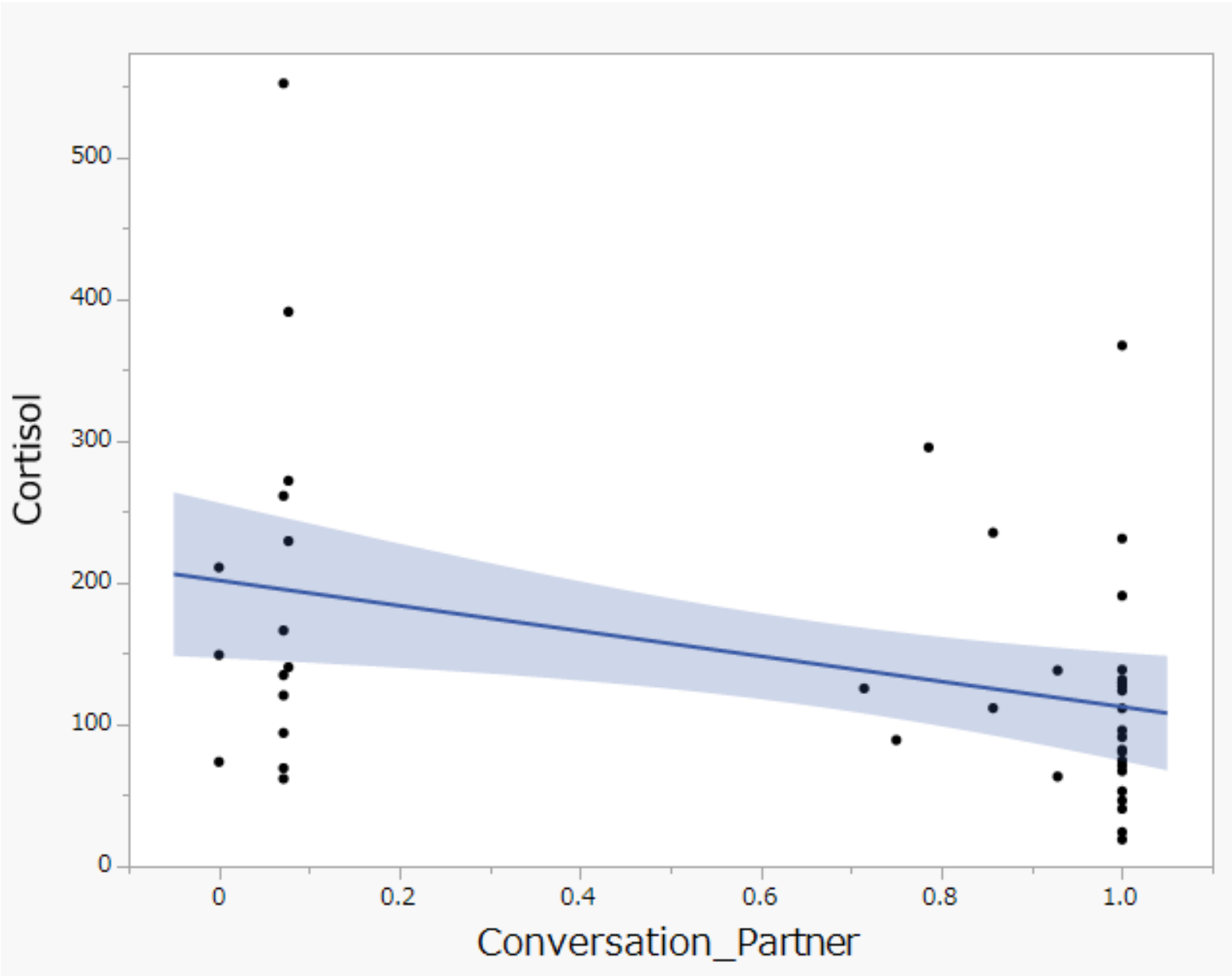

Figure S3. Correlation between conversation with partner and cortisol concentration (ng/mg*cre). The "conversation_partner" shows the proportion of days (out of two weeks) on which conversations with a partner occurred.

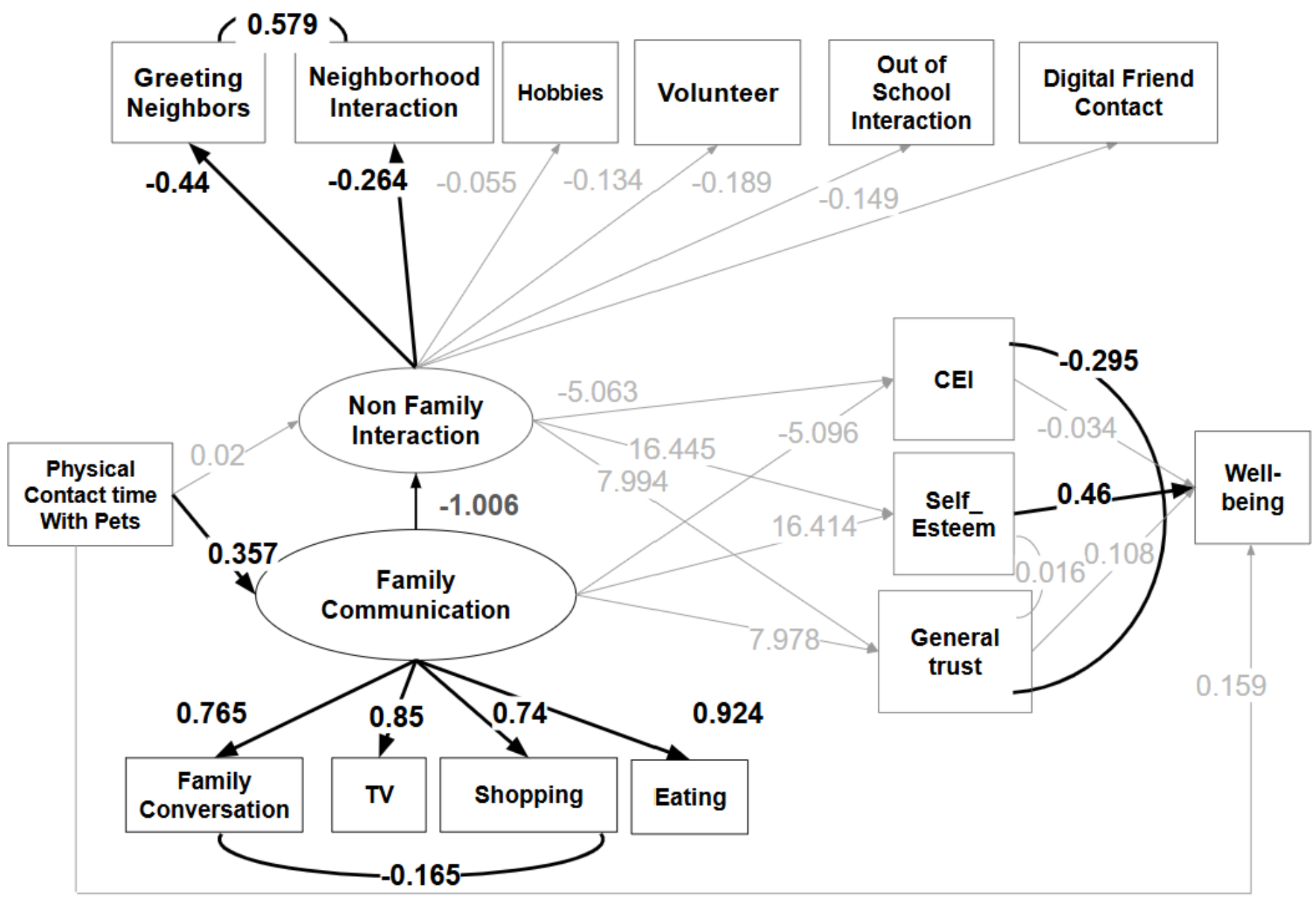


Figure S4. A model of the effects of two-week physical contact time with pets on well-being. The values in the figure represent standardized regression coefficients, factor loadings, or covariances. Boldface indicates significant paths.

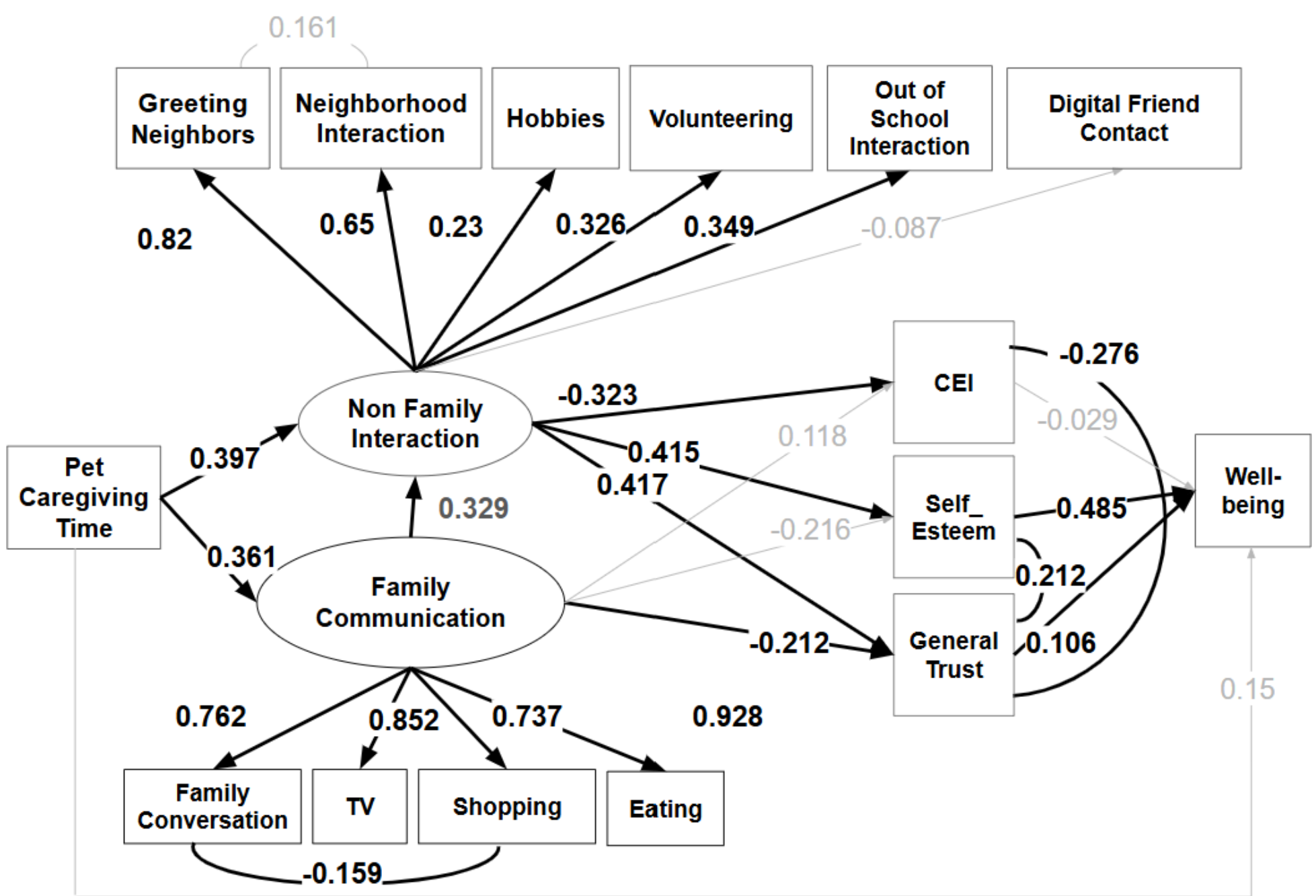


Figure S5. A model of the effects of two-week caregiving time for pets on well-being. The values in the figure represent standardized regression coefficients, factor loadings, or covariances. Boldface indicates significant paths.

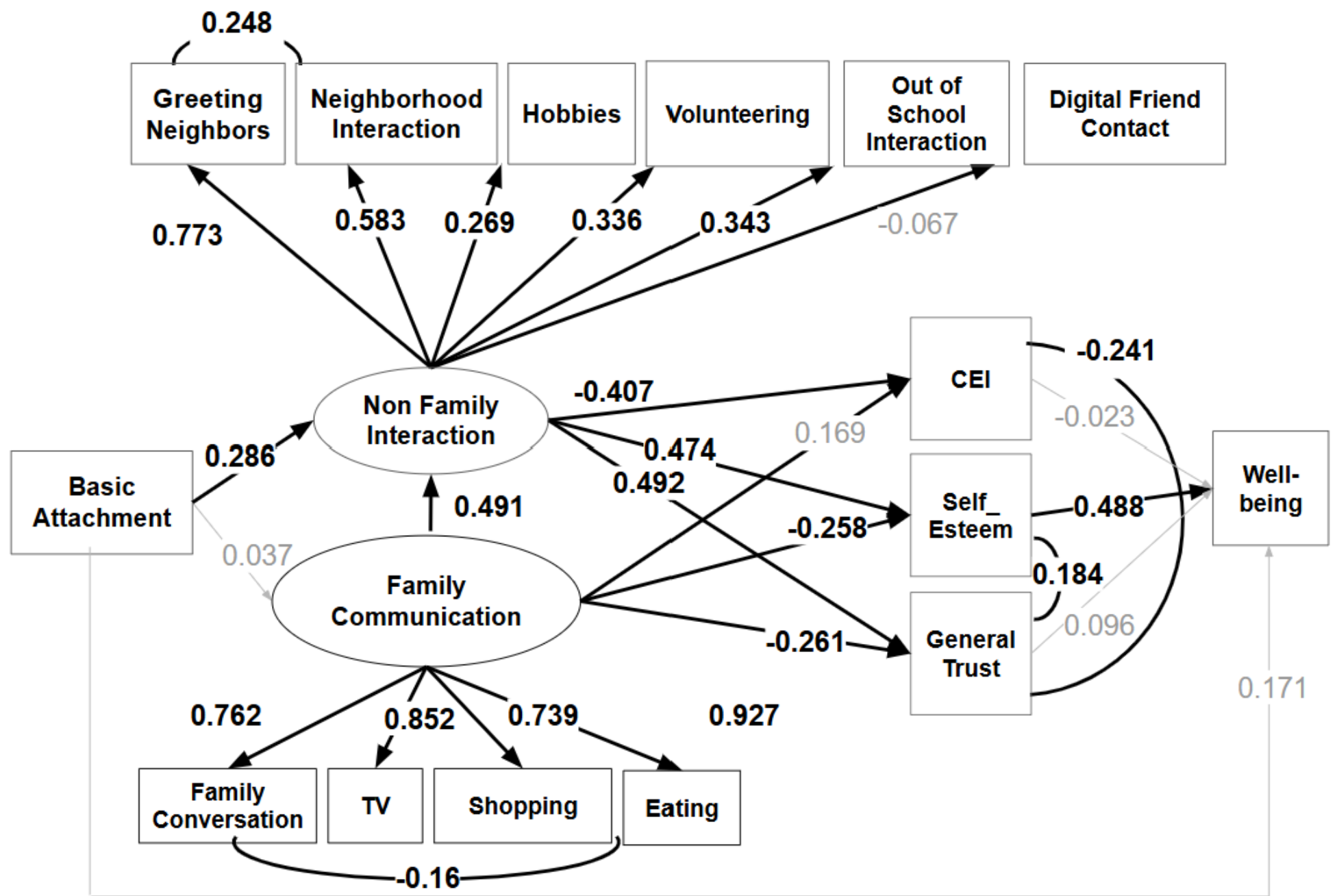


Figure S6. A model of the effects of basic attachment for pets on well-being. The values in the figure represent standardized regression coefficients, factor loadings, or covariances. Boldface indicates significant paths.

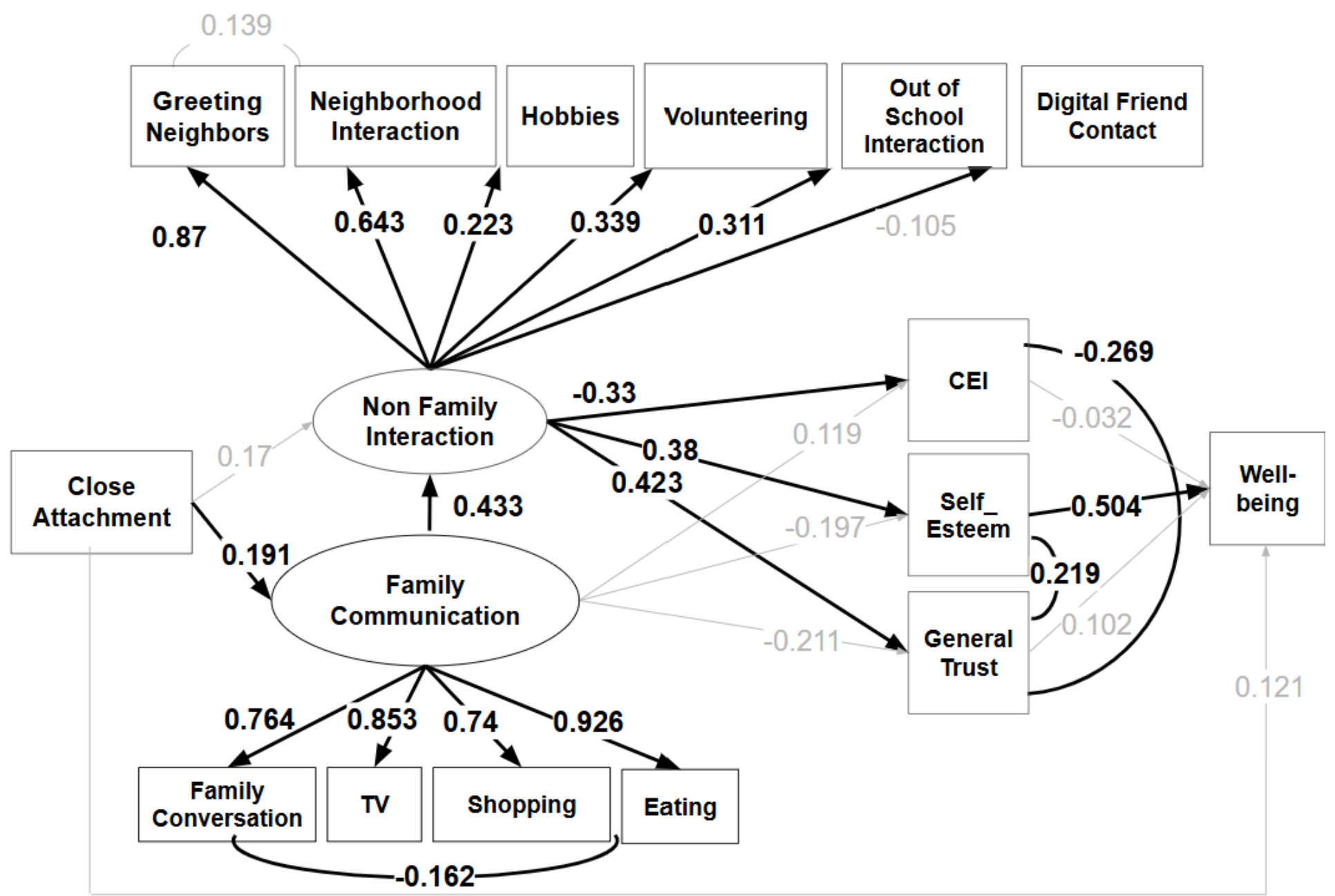


Figure S7. A model of the effects of close attachment for pets on well-being. The values in the figure represent standardized regression coefficients, factor loadings, or covariances. Boldface indicates significant paths.
