# Supplementary material for: Social and biological pathways linking relationships with companion animals to human well-being
Source: Front Psychol. 2026 Sep 3;17:1810355. doi: 10.3389/fpsyg.2026.1810355 (PMC13581753; doi:10.3389/fpsyg.2026.1810355)
Supplement: Supplementary file 2 [file Presentation_2.PDF]

Experience sampling (Daily) questionnaire

Family Conversations

1. Did you spend any time talking with family members yesterday, other than exchanging greetings? (Select all that apply.)

☐ Father
☐ Mother
☐ Sibling(s)
☐ Grandparent(s)
☐ Partner (e.g., husband, wife)
☐ Child(ren)
☐ I did not have an opportunity to talk with family members
☐ Other (please specify: ________)

---

2. Please describe the topics or content of your conversations as much as possible. If you did not have any conversations, please write "No conversation."

Examples:

- I talked with my father about my future career plans.
- I told my mother about something unpleasant that happened at school.
- My brother told me to clean the bathroom, and we ended up arguing.

Shared Activities with Family

3. Yesterday, how many family members (excluding yourself) did you do the following activities with?

If you did not do the activity with anyone, select **0**. If the number exceeds 10, select **10**.

Activity	Number of family members

Watched television, streaming services, or other media content together	0-10
Went shopping together	0-10
Ate out together	0-10
Ate a meal together at home	0-10

Community and Social Interactions

4. Yesterday, how many people did you interact with in the following situations?

If the number exceeds 10, select **10**.

Situation	Number of people
Neighbors with whom you exchanged greetings	0-10
Neighbors with whom you had conversations beyond greetings, worked together, or helped each other	0-10
People you interacted with through sports clubs, lessons, hobbies, or similar activities	0-10
People you interacted with through volunteer or community activities	0-10
People you interacted with through your workplace (including part-time employment)	0-10

Interactions with Close Friends

5. Yesterday, did you engage in the following activities with close friends (friends with whom you can talk about almost anything)?

Please indicate the number of people involved, excluding yourself.

Activity	Number of friends
Contacted them (e.g., LINE, e-mail, phone call)	0-10
Met, went out, or had a meal together in a private setting (outside school or work)	0-10

Interactions with Pets

6. Please indicate your interactions with your pet(s) (dog(s) and/or cat(s)) yesterday.

If you do not own a pet, select **"Do not own a pet."**

Item	Response
Time spent in physical contact with your pet(s) at home	Do not own a pet / 0 min / ≤15 min / ≤30 min / 30 min-1 h / >1 h
Time spent caring for your pet(s)	Do not own a pet / 0 min / ≤15 min / ≤30 min / 30 min-1 h / >1 h

---

7. Please indicate whether the following situations occurred yesterday.

If you do not own a pet, select **"Do not own a pet."**

Item	Response
I interacted with people outside my family through my pet(s).	Yes / No / Do not own a pet

I went out with family members and my pet(s).	Yes / No / Do not own a pet

---

Interactions with Dogs and Cats Other Than One's Own Pets

8. Please indicate your interactions with dogs and/or cats that were not your own pets yesterday.

Item	Response
Interaction with someone else's pet(s)	0 min / ≤15 min / ≤30 min / 30 min-1 h / >1 h
Interaction with dogs and/or cats at a pet café or similar venue	0 min / ≤15 min / ≤30 min / 30 min-1 h / >1 h

---

Lifestyle Variables

9. How many meals did you eat yesterday?

◯ None
◯ One meal
◯ Two meals
◯ Three meals

---

10. Approximately how many hours did you sleep yesterday?

Please indicate the number of hours slept. If you slept more than 10 hours, select 10.

Current Situation

11. Where are you and what are you doing right now?

Please briefly describe your current situation.

Final Questionnaire

Participant Information

1. E-mail Address

2. Name

3. Gender

○ Female
○ Male
○ Other

4. Age

Please enter your age in years.

---

5. Occupation / Educational Status

○ Elementary school student
○ Junior high school student
○ High school student
○ Undergraduate or graduate student
○ Other student (e.g., junior college, vocational school)
○ Employed
○ Other (please specify)

---

6. Including yourself, how many people currently live in your household?

___ persons

---

7. Which family members currently live with you? (Select all that apply.)

☐ Father
☐ Mother
☐ Sibling(s)
☐ Grandparent(s)
☐ Partner (e.g., spouse)
☐ Child(ren)

8. How many close friends do you currently have (friends with whom you can talk a bout almost anything)?

○ 1
○ 2
○ 3
○ 4
○ 5
○ 6
○ 7
○ 8
○ 9
○ 10 or more

Pet Ownership

9. Please indicate your experience of pet ownership.

○ No experience owning pets
○ Experience owning dogs and/or cats (currently or previously)
○ Experience owning other types of pets (currently or previously)

10. Please indicate all that apply.

☐ Dog (currently owned)
☐ Cat (currently owned)
☐ Dog (previously owned, but not currently)
☐ Cat (previously owned, but not currently)

11. Please indicate the breed(s) of your dog(s) and/or cat(s).

Examples: Toy Poodle, Scottish Fold, Mixed breed

Please distinguish between current and past pets.

12. Where were/are your pets primarily kept? (Select all that apply.)

☐ Dog: Free access indoors
☐ Dog: Restricted area indoors
☐ Dog: Outdoors
☐ Cat: Free access indoors
☐ Cat: Restricted area indoors
☐ Cat: Outdoors

---

13. Are there any behaviors of your pet(s) that you consider problematic or difficult to manage?

Please describe them as much as possible.

If none, please write "None."

Pet Attachment Scale

Please select the response that best reflects your opinion.

Response options:

1 = Strongly disagree
2 = Disagree
3 = Neither agree nor disagree
4 = Agree
5 = Strongly agree

14. I feel closer to my pet than to any member of my family.
15. I sometimes think of my pet as my best friend.
16. Being with my pet makes me feel relaxed.
17. My pet makes me feel happy.
18. I would rather not spend time taking care of my pet.
19. Keeping a pet is a waste of money.
20. I often tell my pet important things or share my innermost thoughts with it.
21. Even when I am away from home, I often think about my pet and want to return home quickly.
22. I frequently talk to my pet.
23. I sleep with my pet.
24. I carry photographs of my pet with me.
25. I dress up my pet.

General Trust Scale

Please select the response that best reflects your opinion.

Response options:

1 = Strongly disagree
2 = Disagree
3 = Somewhat disagree
4 = Neither agree nor disagree
5 = Somewhat agree
6 = Agree
7 = Strongly agree

26. Most people are basically honest.
27. I tend to trust other people.
28. Most people are basically good and kind.
29. Most people trust others.
30. Most people are trustworthy.
31. When people are trusted by others, they tend to trust those people in return.

Cultural Estrangement Scale

Please select the response that best reflects your opinion.

Note: “People around you” refers to family members and friends.

Response options:

1 = Strongly disagree
2 = Disagree
3 = Somewhat disagree
4 = Neither agree nor disagree
5 = Somewhat agree
6 = Agree
7 = Strongly agree

32. I feel that my opinions on important matters are typical of, or similar to, those of people around me.
33. I feel that my worldview is consistent with the worldview of people around me.
34. I feel that many of my values are universal and shared by people around me.

35. I feel that I am a good example of what people around me would consider a typical Japanese person.
36. I strongly empathize with the values held by people around me.
37. I feel that most people around me do not understand me.
38. My opinions on important matters tend to differ from those of people around me.
39. I feel that I am very different from what would be considered a “typical person.”
40. I sometimes feel that I do not fit in well with people around me.
41. People sometimes tell me that I am unusual or different.

WHO-5 Well-Being Index

Please indicate how you have felt during the past two weeks.

Response options:

0 = At no time
1 = Some of the time
2 = Less than half of the time
3 = More than half of the time
4 = Most of the time
5 = All of the time

42. I have felt cheerful in good spirits.
43. I have felt calm and relaxed.
44. I have felt active and vigorous.
45. I woke up feeling fresh and rested.
46. My daily life has been filled with things that interest me.

Self-Esteem Scale

Please indicate the response that best describes you.

Response options:

1 = Strongly disagree
2 = Disagree
3 = Agree
4 = Strongly agree

47. On the whole, I am satisfied with myself.
48. At times I think I am no good at all.

49. I feel that I have a number of good qualities.
50. I am able to do things as well as most other people.
51. I feel I do not have much to be proud of.
52. I certainly feel useless at times.
53. I feel that I am a person of worth, at least on an equal basis with others.
54. I wish I could have more respect for myself.
55. All in all, I am inclined to feel that I am a failure.
56. I take a positive attitude toward myself.

Everyday Discrimination Scale

How often have the following experiences occurred in your daily life?

Response options:

1 = Never
2 = Less than once a year
3 = A few times a year
4 = A few times a month
5 = At least once a week
6 = Almost every day

57. You are treated with less courtesy or respect than other people.
58. You receive poorer service than other people at restaurants or stores.
59. People act as if they think you are not intelligent.
60. People act as if they are afraid of you.
61. You are threatened or harassed.

Perceived Reasons for Discrimination

62. What do you think are the main reasons for these experiences? (Select all that apply.)
    - ☐ Ancestry or nationality
    - ☐ Gender
    - ☐ Race
    - ☐ Age
    - ☐ Religion
    - ☐ Height
    - ☐ Weight
    - ☐ Other aspects of physical appearance
    - ☐ Sexual orientation
    - ☐ Educational level or income
    - ☐ Physical disability
    - ☐ Skin color

☐ Ethnicity
☐ No such experiences
☐ Other (please specify: ________)

Health and Lifestyle

63. During the week prior to fecal sample collection, did you consume any supplements, probiotic beverages, yogurt, or similar products?

If yes, please describe the type, amount, and frequency of consumption.

If none, please write "None."

64. During the six months prior to fecal sample collection, did you take any medications, including antibiotics?

If yes, please describe the type, amount, and frequency.

If none, please write "None."

65. Do you have any history of smoking?

If yes, please indicate the frequency.

66. Do you have any history of alcohol consumption?

If yes, please indicate the frequency.

67. Please describe any past or current illnesses, including diagnosis, timing, and symptoms, as much as possible.

If not applicable, please write "None."

68. Please report your Body Mass Index (BMI).

BMI = Weight (kg) ÷ Height $(m)^2$

69. If you have any comments, questions, or concerns regarding this study, please write them below.
